# Performance of estimated glomerular filtration rate equations across ethnic groups in the AIM CKD UK cohort: a multicentre cross-sectional study

**DOI:** 10.1016/j.lanepe.2026.101828

**Published:** 2026-08-25

**Authors:** Rouvick Mariano Gama, Debasish Banerjee, Sarah Crosbie, Kathryn Dalrymple, Sunil Daga, Pierre Delanaye, Ailsa Doak, Leonard Ebah, June Fabian, Darren Green, Sharlene Greenwood, Richard Haynes, Neil Heraghty, Parminder Judge, Shahar Khan, Jennifer Susan Lees, Thomas Lindsay, Nathan Lorde, Rupert Major, Kieran McCafferty, Thomas McDonnell, Sandip Mitra, Javeria Peracha, Adrien Michael Peters, Harshavardhan Sanathkumar, Edward Sharples, Richard Shemilt, John Stoves, Milo To, Michael Turner, Aniebiotabas Udofia, Paul Cockwell, Kate Bramham

**Affiliations:** a Department of Inflammation Biology, Faculty of Life Sciences and Medicine, King's College London, London, United Kingdom; b King's Kidney Care, King's College Hospital, King's College Hospital NHS Foundation Trust, Denmark Hill, London, United Kingdom; c Renal and transplant unit, St George's Hospital, St George's University Hospitals NHS Foundation Trust, London, United Kingdom; d Department of Medicine, City St George's University of London, London, United Kingdom; e Oxford Kidney Unit, Churchill Hospital, Oxford University Hospitals NHS Foundation Trust, London, United Kingdom; f Department of Nutritional Sciences, Faculty of Life Sciences and Medicine, King's College London, London, United Kingdom; g Institute of Health Science, University of Leeds, Leeds, United Kingdom; h Renal Research Department, St James's Hospital, Leeds Teaching Hospitals NHS Trust, Harehills, Leeds, United Kingdom; i Department of Nephrology-Dialysis-Transplantation, University of Liege, Liege, Belgium; j Renal Department, St Luke's Hospital, Bradford Teaching Hospitals NHS Foundation Trust, Bradford, United Kingdom; k Manchester institute for nephrology and transplantation (MINT) Laboratories, Manchester Royal Infirmary, Manchester University NHS Foundation Trust, Manchester, United Kingdom; l Wits Donald Gordon Medical Research Institute, Faculty of Health Sciences, University of the Witwatersrand, 27 Eton Rd, Parktown, Johannesburg, South Africa; m Department of Nephrology, Salford Royal Hospital, Northern Care Alliance, Salford, United Kingdom; n Renal Studies Group, Clinical Trials Service Unit, University of Oxford, Oxford, United Kingdom; o Department of Nuclear Medicine, King's College Hospital, King's College Hospital NHS Foundation Trust, Denmark Hill, London, United Kingdom; p Barts Health Renal Department, Royal London Hospital, Barts Health NHS Trust, London, United Kingdom; q Glasgow Renal and Transplant Unit, Queen Elizabeth University Hospital, London, United Kingdom; r BHF Cardiovascular Research Centre, University of Glasgow, Glasgow, United Kingdom; s Department of Biochemistry, University Hospitals Birmingham NHS Foundation Trust, Birmingham, United Kingdom; t Renal Department, Leicester General Hospital, University Hospitals of Leicester, Leicester, United Kingdom; u Social science, Applied Healthcare and Improvement Research, University of Leicester, Leicester, United Kingdom; v Renal Department, University Hospitals Birmingham NHS Foundation Trust, Birmingham, United Kingdom

**Keywords:** Creatinine, Glomerular filtration rate, eGFR, mGFR, Ethnicity, CKD-EPI, EKFC

## Abstract

**Background:**

Accurate assessment of kidney function using estimated glomerular filtration rate (eGFR) is fundamental for chronic kidney disease diagnosis and management, although performance is variable in African and Asian cohorts. We evaluated creatinine-based eGFR performance in an ethnically diverse United Kingdom cohort.

**Methods:**

We conducted a multi-centre, cross-sectional study. We included adults who had paired measured GFR (mGFR) and serum creatinine tests between 2009 and 2022. eGFR was calculated using five validated creatinine-based equations. The primary outcome was eGFR agreement with mGFR determined by assessing correlation, difference, precision and 30% accuracy (P30) with stratification by ethnicity.

**Findings:**

The study included 15,879 participants (mean age 54 ± 16 years; 8893/15,879 (56%) male). On average, all equations overestimated mGFR. There were significant variations in difference (p < 0.001) and accuracy (p < 0.001) between all equations. Lund-Malmo Revised (LMR) had the least difference (1.4 mL/min/1.73 m^2^; 95% CI: 1.1–1.6 mL/min/1.73 m^2^) and highest accuracy (P30: 86.0%; 95% CI: 85.5–86.6%), followed by European Kidney Function Consortium (EKFC).

eGFR had the greatest difference and lowest accuracy in South Asians, compared with other ethnicity groups. mGFR was substantially overestimated in young adults (<25 years), low BMI (<20 kg/m^2^) and hypoalbuminaemia (<20 g/L). Hypothetical prescribing analyses for dapagliflozin (upper GFR threshold: 75 mL/min/1.73 m^2^) showed potential prescribing ineligibility based on eGFR alone in 29% (1933/6586) to 52% (3433/6586) of eligible participants.

**Interpretation:**

LMR and EKFC showed improved agreement with mGFR, particularly in South Asians and young adults. Further prospective validation is recommended to support changes in clinical practice.

**Funding:**

Kidney Research UK.

**Research in context**

**Evidence before this study**

We searched PubMed and Embase for original studies published between 1 January 2009 and 31 December 2025 evaluating creatinine-based estimated glomerular filtration rate (eGFR) equations against measured GFR (mGFR) in ethnically diverse adult populations. Searches were not restricted by language. Search terms included combinations of “estimated glomerular filtration rate”, “eGFR”, “measured GFR”, “mGFR”, “CKD-EPI”, “MDRD”, “EKFC”, “Lund-Malmo”, “ethnicity”, “race”, “Black”, “African”, “Asian”, and “South Asian”.

Existing evidence showed variable performance of creatinine-based eGFR equations across ethnicity groups. The Chronic Kidney Disease Epidemiology Collaboration (CKD-EPI) 2021 equation, developed in the USA as a race-neutral alternative, reported P30 accuracy of 78.3–86.5% in White and Black American cohorts, but lower accuracy in East and South African and Indian cohorts. The revised Lund-Malmo (LMR) and European Kidney Function Consortium (EKFC) equations, developed in European cohorts, have reported P30 accuracy above 85% in White European populations and have shown improved performance compared with CKD-EPI in some African and South Asian cohorts. Risk of bias was moderate with substantial heterogeneity in study design, study populations, mGFR protocols and analyte assays. However, eGFR equation performance in ethnically diverse UK populations remains uncertain, reinforced by National Institute for Health and Care Excellence (NICE) recommending further validation in ethnic minority and young adult and paediatric populations.

**Added value of this study**

This is one of the largest studies worldwide, and the largest South Asian cohort, to directly compare pre-selected creatinine-based eGFR equations against mGFR. Our study shows that all creatinine-based eGFR equations overestimate mGFR on average, and the widely used CKD-EPI equations are the lowest performing across all ethnicities. eGFR equations showed the highest difference and lower accuracy of eGFR equations in people from South Asian backgrounds compared to other ethnicity groups. The LMR and EKFC equations consistently performed better with lower difference and higher 30% accuracy across ethnicities, as well as subgroups such as young adults and cohorts with expected altered body composition.

**Implications of all the available evidence**

Our findings suggest that CKD prevalence may be underestimated, particularly in ethnic minority groups, young adults and those with altered body composition, which has the potential to impact diagnosis and prescribing practices in selected populations. Taken together, these findings highlight the potential of the LMR and/or EKFC equations to improve accurate assessment of kidney function, particularly in high-risk groups. However, there remains a need for regional validation data before widespread adoption.

## Introduction

Chronic kidney disease (CKD) affects approximately 10% of the global population and is projected to increase in prevalence.^1^ CKD has substantial health and economic impacts on healthcare systems leading to adverse outcomes including kidney failure, cardiovascular events, and premature death.^2^

The 2024 international guidelines by Kidney Disease: Improving Global Outcomes (KDIGO) recommend using glomerular filtration rate (GFR) assessment to diagnose and stage CKD, estimate kidney failure risk and guide prescribing of renally-excreted medications.^3^ Measured GFR (mGFR) is the reference standard for kidney function assessment but is impractical for routine clinical practice because it is time-consuming, costly, and requires serial venous sampling. Therefore estimated GFR (eGFR), calculated from serum creatinine, age, and sex, is commonly used. Accurate eGFR measurement is essential for diagnosis, specialist care referral, management, and prognosis of CKD and has become increasingly important with licencing of new cardiorenal protective therapies with eGFR thresholds for initiation.^4^

The Modified Diet in Renal Disease (MDRD) and Chronic Kidney Disease Epidemiology Collaboration (CKD-EPI) 2009 equations, which were developed predominantly from cohorts in the USA, are most widely used globally.^5^^,^^6^ However, these equations have shown reduced accuracy in African and South Asian populations, with systematic overestimation of measured GFR (mGFR).^7^^,^^8^ Newer eGFR equations from Europe and ‘ethnicity-free’ equations from the USA have now been developed with gradual rise in implementation.^9^^,^^10^ However, ethnicity is a social construct that varies across geographies, therefore assessment of eGFR equation performance in diverse cohorts is required to determine suitability before adoption in clinical practice.

As one of Europe's most ethnically diverse countries, the United Kingdom's (UK) system of universal healthcare and permitted recording of reported ethnicity, provides a unique opportunity to use routinely collected data to study diverse populations with sufficient power to inform practice.^11^ The National Institute for Health and Care Excellence (NICE) 2021 CKD guidelines recommended further research to evaluate performance of eGFR equations in young adults and ethnic minority groups.^12^

AIM CKD UK (Assessing and Improving creatinine-based eGFR to evaluate kidney disease in the UK) aims were to (1) evaluate performance of creatinine-based eGFR equations compared to mGFR in multiple self-reported ethnic groups, (2) identify those in whom creatinine-based eGFR equations are suboptimal and (3) describe the impact of different eGFR equations on prescribing practices for medications with eGFR prescribing thresholds routinely used in CKD care.

## Methods

### Study design

We performed a retrospective observational multi-centre cross-sectional study (Supplementary Figure S1). Eleven sites with high proportions of people with kidney disease from non-White ethnicities were selected. Inclusion criteria were adults (≥18 years) with a mGFR test using plasma clearance of Chromium-51 labelled ethylenediaminetetraacetate (^51^Chromium-EDTA) or Technetium-99m labelled diethylenetriamine pentaacetate (^99m^Technetium-DTPA) and a paired isotopic dilution mass spectrometry (IDMS)-traceable serum creatinine (Jaffe or Enzymatic assay) within 30 days. Data were collected for all individuals, who met the inclusion criteria between 1st January 2009 and 31st December 2022. Exclusion criteria were children, missing age or sex, national data opt-out, and mGFR>150 mL/min/1.73 m^2^ (considered physiologically implausible in adults).^13^

### Data collection

Age, sex, height, weight, time of mGFR and creatinine tests, mGFR adjusted for normalised body surface area, mGFR filtration marker, IDMS-traceable serum creatinine concentration, creatinine assay, serum albumin concentration, referral speciality and ethnicity were extracted retrospectively from pre-existing clinical electronic health records at each individual site. Ethnicity was recorded according to National Census categories and grouped as White, Black, South Asian, East Asian and ‘Other or Not Specified’. Grouping of National Census categories is included in Supplementary Table S1.

eGFR was calculated using five creatinine-based equations (Supplementary Table S2): MDRD without ethnicity coefficient (MDRD-3v), CKD-EPI 2009 without ethnicity coefficient (CKD-EPI_2009_), CKD-EPI 2021 ‘race-neutral’ (CKD-EPI_2021_), European Kidney Function Consortium (EKFC) and Lund-Malmo Revised (LMR) equations.^5^^,^^6^^,^^9^^,^^10^^,^^14^ These equations were chosen due to being directly relevant to recent, current or potentially future UK/European clinical practice and are all included in the KDIGO CKD Guidelines 2024, except MDRD and LMR.^3^ Detailed rationale for including these equations is included in the Supplementary Methodology.

mGFR was extracted retrospectively from electronic health records. mGFR studies were performed as per local routine clinical practice and therefore were not specific to this study. Further detail on mGFR testing is provided in the Supplementary Methodology: Measured glomerular filtration rate. The primary outcome was based on clinically reported NHS laboratory and physiology measurements used in routine care, while demographic and referral variables were extracted from routinely coded electronic health records.

### Outcomes

The primary outcome was agreement between eGFR equations and mGFR stratified by ethnicity groups. Agreement was measured using difference (eGFR minus mGFR; also referred to as ‘bias’ in GFR evaluation studies), precision (standard deviation or IQR of the difference), Lin's concordance correlation coefficient, Bland–Altman plots with 95% limits of agreement and accuracy. P30 was used for accuracy which is the proportion of eGFR results which fall within 30% of the corresponding mGFR value. This balances achievable accuracy with clinical relevance and is a recognised international standard for assessing GFR performance, with KDIGO 2024 CKD Guidelines recommending ≥80% as a clinically acceptable threshold and above 90% as optimal.^3^ For mean difference, median difference and P30, 95% confidence intervals (CI) were calculated using the t-distribution, a distribution-free order statistic method, and the normal approximation to the binomial proportion respectively.

Secondary outcomes included agreement between eGFR and mGFR stratified by age, body mass index (BMI), sex, albumin, referral speciality, proportion of misclassification of CKD staging (KDIGO thresholds but with single GFR) for each equation. The hypothetical impact of prescribing thresholds for commonly used medications was estimated for each equation.

### Sample size

We calculated a required sample size of 1200 participants (240 participants per group), to detect a significant difference in eGFR-mGFR difference and 30% accuracy between five ethnicity groups, based on an alpha level of 0.05, 80% power, small effect size (Cohen's f = 0.10), and assumed equal group variances. A post hoc power analysis was performed using the observed sample sizes, with clinically meaningful thresholds defined as a difference between eGFR and mGFR of 5 mL/min/1.73 m^2^ assessed using a one sample t-test for each ethnicity group independently. This value was defined as the minimum clinically relevant threshold, based on similar designed studies in the literature defining bias or difference as 3–5 mL/min/1.73 m^2^.^11^ Power required to detect a 5% difference in P30 between the five ethnicity groups was assessed using a chi-squared test, with an alpha level of 0.05 and 80% power threshold applied throughout.

### Statistical analysis

Categorical data were presented as counts and percentages; numerical data were presented according to distribution. Missing data were handled using a complete case approach for essential variables (mGFR, age, sex, creatinine). Participants with missing non-essential variables were excluded from relevant sub-analyses but were retained for the overall cohort.

We reported difference as mean and median with 95% confidence intervals (95% CI). Precision was reported as standard deviation (SD) and interquartile range (IQR) of the difference. Accuracy was reported as P30 percentage with 95% CI and root mean square error (RMSE). 95% limits of agreement were illustrated using Bland Altman plots.^15^

We compared difference between eGFR equations using repeated-measure ANOVA tests with post hoc analysis using paired t-test with a Bonferroni correction. Assumptions of normality were assessed using Q–Q plots; Mauchly's test assessed sphericity, with Greenhouse-Geisser correction if sphericity was violated. P30 was compared using Chi-Squared tests, with Fisher tests for post hoc analyses. Analyses were repeated with stratification for ethnicity groups; difference was compared using a one-way ANOVA and Tukey HSD post-hoc.

We used multivariable linear regression modelling to examine associations between eGFR bias (outcome) and ethnicity group (predictor). Demographic (age, sex, and BMI), clinical (serum albumin concentration and referral speciality) and analytical (creatinine assay; time between mGFR and eGFR tests and mGFR filtration marker) variables were included in the model as potential confounders to assess the association on eGFR difference and ethnicity group. Model assumptions were assessed separately for each eGFR equation model. Linearity was assessed using residuals vs fitted plots, normality of residuals using Q–Q plots, homoscedasticity using scale-location plots and leverage/influence using residual vs leverage plots with Cook's distance. No assumption violations were identified that materially altered interpretation of the regression analyses.

We visualised secondary continuous variables (e.g. age) using scatter plots with locally weighted scatterplot smoothing (LOWESS) lines for difference and bar charts for P30. Categorical variables (e.g. referral speciality) were visualised using faceted density plots to demonstrate distribution of difference and bar charts for P30.

Correct classification of KDIGO CKD Stage (eGFR stage aligned with mGFR stage) was reported for each eGFR equation as a proportion (percentage) with 95% CI.

Potential impact of eGFR error on prescribing of commonly used medications (SGLT2i, Angiotensin-converting enzyme inhibitors (ACEi), Fineronone and metformin) with eGFR initiation thresholds was evaluated (Supplementary Table S3) using proportions with 95% CI of those eligible and ineligible according to each mGFR threshold. Analyses were performed using R Software version 4.4.2.

### Patient and public involvement

Patient and public involvement was incorporated in the development of the AIM CKD UK study, with input from Kidney Care UK Patient Advisory Group alongside engagement from the South Asian Health Foundation and Africa Advocacy Foundation. Patient representatives agreed on the importance of evaluating eGFR across diverse ethnic groups and emphasised the need to translate findings into meaningful outcomes for patients. These included the potential impact on CKD diagnosis, staging classification and prescribing decisions, for medications dependent on kidney function eGFR thresholds.

### Ethical approval

The study protocol received favourable opinion from the UK Health Research Authority on 17th April 2023 (23/PR/0324).

### Role of the funding source

Kidney Research UK provided funding for the study but had no role in study design, data collection, data analysis, data interpretation, writing the manuscript, or decision to submit for publication.

## Results

### Baseline characteristics

The study included 15,879 participants who met inclusion criteria between 2009 and 2022. Demographic and renal characteristics are summarised in Table 1. Mean age was 54.0 ± 15.7 years, 8893 were male (56%) and median BMI was 26.1 kg/m^2^ (IQR 23.0, 29.8 kg/m^2^). White ethnicity comprised the largest group (n = 9885; 62.3%), followed by ‘Other or Not Specified’ (n = 4195; 26.4%), South Asians (n = 891; 5.6%), Black (n = 840; 5.3%) and East Asian ethnicities (n = 68; 0.4%).

**Table 1:** Demographic and renal characteristics of the cohort stratified by high-level ethnicity groups.

Variable	Whole cohort	Ethnicity group				
		White	Black	South Asian	East Asian	Other/Not stated
Number of participants (N)	15,879	9885	840	891	68	4195
Sex, N (%):						
Male	8893 (56.0)	5493 (55.6)	446 (53.1)	447 (50.2)	45 (66.2)	2462 (58.7)
Female	6986 (44.0)	4392 (44.4)	394 (46.9)	444 (49.8)	23 (33.8)	1733 (41.3)
Mean Age ± SD, years	54.0 ± 15.7	55.0 ± 15.6	55.4 ± 14.8	49.2 ± 16.1	57.1 ± 15.8	52.5 ± 15.8
Median BMI (IQR), kg/m^2^	26.1 (23.0–29.8)	26.3 (23.1–30.0)	26.9 (23.4–31.1)	26.2 (23.2–29.2)	22.1 (20.0–24.5)	25.6 (22.9–29.4)
Median creatinine (IQR), *μ*mol/L	75 (63–92)	75 (63–92)	81 (66–101)	72 (59–92)	72 (61–92)	74 (62–90)
Mean mGFR corrected for standardised BSA ± SD, mL/min/1.73 m^2^	77.1 ± 24.0	76.2 ± 23.9	74.7 ± 22.4	75.5 ± 26.7	82.7 ± 28.2	80.0 ± 23.4
CKD, (mGFR <60 mL/min/1.73 m^2^), N (%)	3648 (23.0)	2377 (24.0)	203 (24.1)	228 (25.6)	16 (23.5)	824 (19.6)
mGFR categories, mL/min/1.73 m^2^, N (%):						
≥90	5100 (32.1)	3021 (30.6)	215 (25.6)	274 (30.8)	25 (36.8)	1565 (37.3)
60–89	7131 (44.9)	4487 (45.4)	422 (50.2)	389 (43.7)	27 (39.7)	1806 (43.1)
30–59	3105 (19.6)	2029 (20.5)	173 (20.6)	167 (18.7)	14 (20.6)	722 (17.2)
15–29	476 (3.0)	313 (3.17)	28 (3.3)	39 (4.4)	2 (2.9)	94 (2.2)
<15	67 (0.4)	35 (0.4)	2 (0.2)	22 (2.5)	0	8 (0.2)
Median days between mGFR and eGFR test (IQR)	0 (0–6)	0 (0–6)	0 (0–7)	0 (0–5)	0 (0–4)	0 (0–7)
Referral speciality, N (%):						
Haematology/Oncology	9799 (61.7)	6195 (62.7)	532 (63.3)	433 (48.6)	49 (72.1)	2590 (61.7)
Nephrology	3116 (19.6)	1858 (18.8)	133 (15.8)	218 (24.5)	7 (10.3)	900 (21.5)
Hepatology	365 (2.3)	219 (2.2)	21 (2.5)	18 (2.0)	1 (1.5)	106 (2.5)
Other/Not stated	2599 (16.4)	1613 (16.3)	154 (18.3)	222 (24.9)	11 (16.2)	599 (14.3)

Mean mGFR was 77.1 ± 24.0 mL/min/1.73 m^2^ including 3648 (23.0%) participants who had mGFR <60 mL/min/1.73 m^2^. Choice of mGFR filtration marker was not reported in 868 cases (5.5%); ^99m^Technetium-DTPA (n = 6465; 40.7%) or ^51^Chromium-EDTA (n = 8546; 53.8%) were otherwise used. Most participants were referred by haematology or oncology services (n = 9799; 61.7%) followed by nephrology services (n = 3116; 19.6%).

Participant characteristics were broadly stable across study time intervals, although the proportion of patients from White ethnic backgrounds gradually decreased from 67.8% in 2009–2011 to 57.1% in 2021–2022. In contrast, the proportion with Other or Not Stated ethnic backgrounds increased from 23.8% to 29.0% across the same time periods (Supplementary Table S4). Referral speciality also changed over time, with nephrology referrals decreasing from 28.2% to 15.6% and Other or Not Stated referrals increasing from 7.2% to 22.5% across the same time periods stated previously.

### Creatinine-based eGFR equation performance

All eGFR equations overestimated mGFR. LMR had least difference (1.35; 95% CI: 1.1–1.6 mL/min/1.73 m^2^) and CKD-EPI_2021_ had greatest difference (13.3; 95% CI: 13.0–13.5 mL/min/1.73 m^2^). Differences for all eGFR equations were statistically significantly different to each other (p < 0.001). Precision was narrowest with the EKFC equation (SD: 14.8 mL/min/1.73 m^2^), and widest with the MDRD-3v (SD: 23.1 mL/min/1.73 m^2^).

Assumptionsfor the repeated-measures ANOVA is illustrated in Supplementary Figure S2. Mauchly's test indicated violation of sphericity. Greenhouse-Geisser corrected analyses remained statistically significant (p < 0.001). Non-parametric sensitivity analysis using Friedman tests with Nemenyi post-hoc comparisons did not materially alter the findings.

LMR had highest accuracy (P30 = 86.0%; 95% CI: 85.5–86.6%), followed by EKFC (83.6%; 95% CI: 83.0–84.2%), CKD-EPI_2009_ (76.0%; 95% CI: 75.4–76.7%), MDRD-3v (73.5%; 95% CI: 72.9–74.2%) and CKD-EPI_2021_ (70.1%; 95% CI: 69.4–70.8%). There were significant differences in accuracy between each equation (each comparison: p < 0.001).

**Fig. 1:**
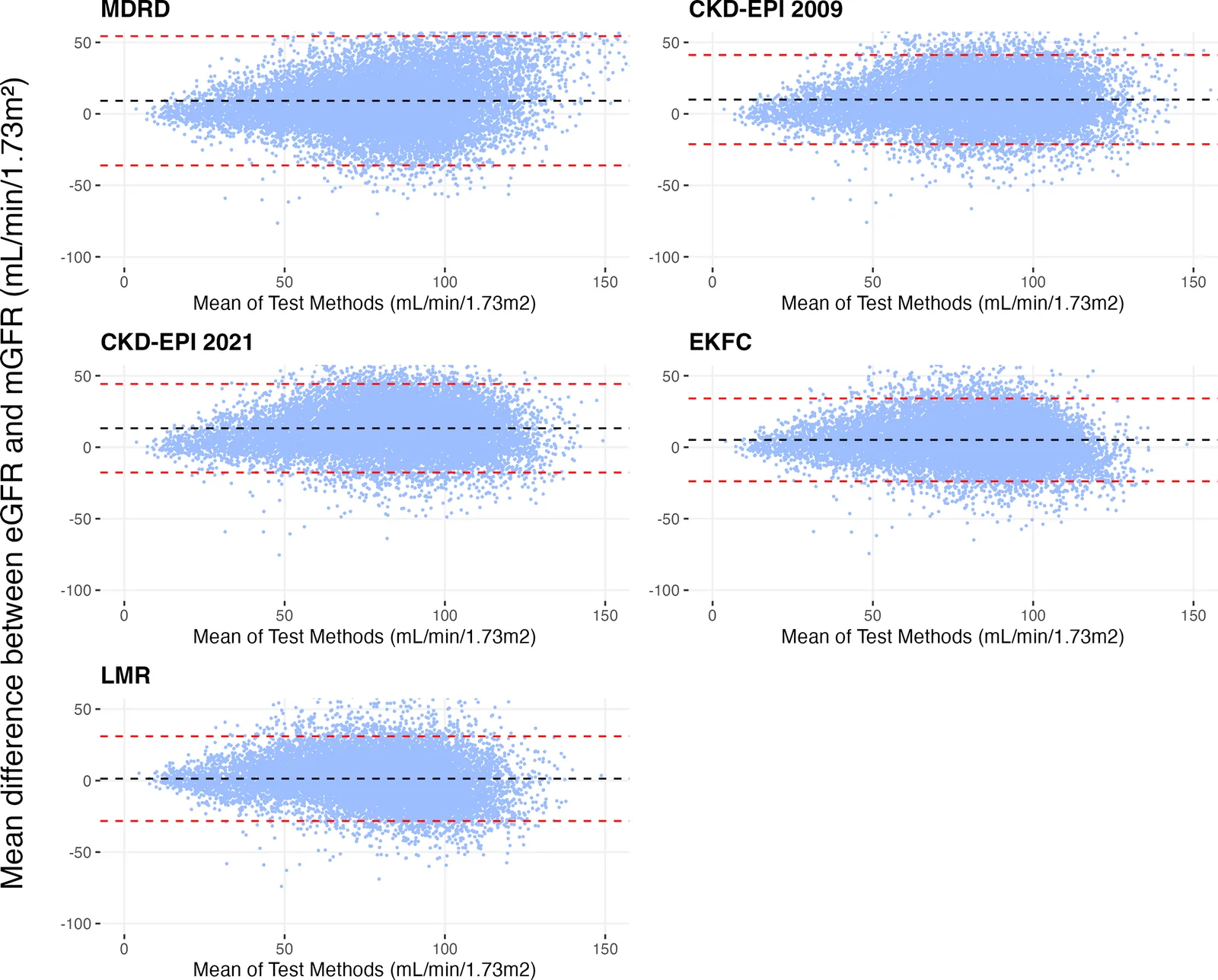
Bland Altman plots demonstrating agreement between each eGFR equation and mGFR with 95% limits of agreement.

Limits of agreement were wide across all equations (Table 2). The LMR had narrowest 95% limits at −28.3 to 31.0 mL/min/1.73 m^2^ whilst the MDRD-3v had the widest limits ranging from −36.1 to 54.4 mL/min/1.73 m^2^ (Fig. 1).

**Table 2:** Difference, precision and accuracy (P30) of the eGFR equations compared to mGFR with stratification by ethnicity.

eGFR equation	Mean difference, mL/min/1.73 m^2^ [95% CI]	SD	Median difference, mL/min/1.73 m^2^ [95% CI]	IQR, mL/min/1.73 m^2^	RMSE, mL/min/1.73 m^2^	P30, % [95% CI]	95% LoA (lower to upper)
Whole Cohort (n = 15,879)							
MDRD-3v	9.1 [ 8.8–9.5]	23.1	6.2 [5.8–6.5]	−4.7 to 19.8	24.8	73.5 [72.9–74.2]	−36.1 to 54.4
CKD-EPI_2009_	10.0 [9.7–10.2]	15.9	9.7 [9.3–9.9]	−0.2 to 19.9	18.8	76.0 [75.4–76.7]	−21.2 to 41.2
CKD-EPI_2021_	13.3 [13.0–13.5]	15.8	13.1 [12.8–13.4]	3.1–23.3	20.6	70.1 [69.4–70.8]	−17.7 to 44.2
EKFC	5.1 [4.9–5.4]	14.8	4.8 [4.6–5.1]	−4.1 to 14.3	15.7	83.6 [83.0–84.2]	−23.8 to 34.1
LMR	1.4 [1.1–1.6]	15.1	1.2 [ 0.9–1.4]	−8.0 to 10.5	15.2	86.0 [85.5–86.6]	−28.3 to 31.0
White ethnicity (n = 9885)							
MDRD-3v	9.4 [9.0–9.8]	22.5	6.2 [5.7–6.6]	−4.1 to 19.4	24.4	74.3 [73.4–75.2]	−34.7 to 53.5
CKD-EPI_2009_	10.1 [9.8–10.4]	15.4	9.6 [9.2–9.9]	0.2–19.6	18.4	76.9 [76.0–77.7]	−20.1 to 40.2
CKD-EPI_2021_	13.4 [13.1–13.7]	15.3	13.2 [12.7–13.5]	3.5–23.1	20.3	70.7 [69.8–71.6]	−16.5 to 43.3
EKFC	5.2 [4.9–5.4]	14.3	4.7 [4.4–5.0]	−3.7 to 13.9	15.2	84.6 [83.8–85.3]	−22.9 to 33.2
LMR	1.5 [1.2–1.8]	14.6	1.2 [0.9–1.5]	−7.5 to 10.3	14.7	86.9 [86.2–87.5]	−27.2 to 30.2
Black ethnicity (n = 840)							
MDRD-3v	2.8 [1.3–4.3]	22.2	0.2 [−1.5 to 1.7]	−11.1 to 15.3	22.3	74.1 [71.1–77.0]	−40.7 to 46.3
CKD-EPI_2009_	4.5 [3.3–5.7]	17.9	3.3 [2.0–5.2]	−6.6 to 16.1	18.5	77.3 [74.4–80.1]	−30.6 to 39.7
CKD-EPI_2021_	7.9 [6.7–9.1]	18.0	6.9 [5.2–8.3]	−3.4 to 19.8	19.6	74.1 [71.1–77.0]	−27.3 to 43.1
EKFC	0.6 [−0.5 to 1.7]	16.4	−0.5 [−1.4 to 0.8]	−9.4 to 10.1	16.4	82.4 [79.8–85.0]	−31.6 to 32.8
LMR	−2.6 [−3.7 to −1.5]	16.3	−3.5 [−4.4 to −2.3]	−12.8 to 6.8	16.5	82.7 [80.2–85.3]	−34.5 to 29.2
South Asian ethnicity (n = 891)							
MDRD-3v	13.5 [11.9–15.1]	24.4	10.6 [9.0–11.8]	−0.9 to 24.9	27.9	64.1 [60.9–67.2]	−34.3 to 61.3
CKD-EPI_2009_	14.8 [13.7–16.0]	17.2	14.6 [13.3–15.7]	3.9–25.5	22.7	65.0 [61.9–68.1]	−18.8 to 48.4
CKD-EPI_2021_	17.7 [16.6–18.8]	16.9	17.8 [16.6–18.9]	6.7–28.7	24.5	58.3 [55.1–61.6]	−15.5 to 50.9
EKFC	9.9 [8.9–11.0]	15.7	10.1 [8.9–11.4]	0.6–19.1	18.6	73.1 [70.2–76.0]	−20.8 to 40.7
LMR	5.7 [4.7–6.8]	15.8	5.8 [4.4–7.1]	−3.5 to 14.9	16.8	78.7 [76.0–81.4]	−25.3 to 36.7
East Asian ethnicity (n = 68)							
MDRD-3v	8.0 [3.0–13.0]	20.6	8.7 [0.5–13.2]	−3.8 to 18.3	22.0	75.0 [64.7–85.3]	−32.4 to 48.5
CKD-EPI_2009_	5.8 [1.8–9.7]	16.2	6.2 [3.2–9.9]	−2.2 to 15.2	17.1	80.9 [71.5–90.2]	−26.1 to 37.6
CKD-EPI_2021_	9.0 [5.1–12.9]	16.1	8.5 [6.6–11.6]	1.1–19.2	18.3	78.0 [68.1–87.8]	−22.6 to 40.6
EKFC	0.4 [−3.4 to 4.1]	15.4	0.6 [−3.9 to 4.6]	−10.9 to 9.1	15.3	82.4 [73.3–91.4]	−29.8 to 30.6
LMR	−3.0 [−6.8 to 0.8]	15.7	−1.5 [−7.1 to 1.6]	−12.5 to 6.1	15.8	85.3 [76.9–93.7]	−33.7 to 27.7
Other/Not Stated ethnicity groups (n = 4195)							
MDRD-3v	8.9 [8.1–9.6]	24.0	6.2 [5.5–6.9]	−6.0 to 20.2	25.6	73.6 [72.3–75.0]	−38.2 to 55.9
CKD-EPI_2009_	9.9 [9.4–10.4]	16.2	10.0 [9.3–10.5]	−0.8 to 20.1	18.9	76.0 [74.7–77.3]	−21.8 to 41.5
CKD-EPI_2021_	13.1 [12.6–13.6]	16.0	13.2 [12.7–13.8]	2.7–23.5	20.7	70.5 [69.1–71.9]	−18.2 to 44.5
EKFC	5.0 [4.6–5.5]	15.0	5.1 [4.5–5.6]	−4.7 to 14.4	15.8	83.8 [82.7–84.9]	−24.4 to 34.4
LMR	0.9 [0.4–1.4]	15.6	0.9 [0.2–1.4]	−8.9 to 10.6	15.6	86.4 [85.3–87.4]	−29.6 to 31.4

When stratified by mGFR CKD stage (Supplementary Table S5), difference increased with advancing stages across all eGFR equations. Performance was best in those with mGFR >90 mL/min/1.73 m^2^. However, with declining mGFR, difference and accuracy were attenuated with the LMR and EKFC equations compared to the MDRD-3v and CKD-EPI equations. There was also variability in difference and accuracy depending on site (Supplementary Table S6).

Equation performance varied across time intervals but the overall pattern remained consistent. CKD-EPI_2021_ demonstrated the greatest positive mean difference and consistently had the lowest P30. Whereas LMR typically had the lowest mean difference and the highest P30 across time periods (Supplementary Table S4).

### Performance in ethnicity subgroups

**Fig. 2:**
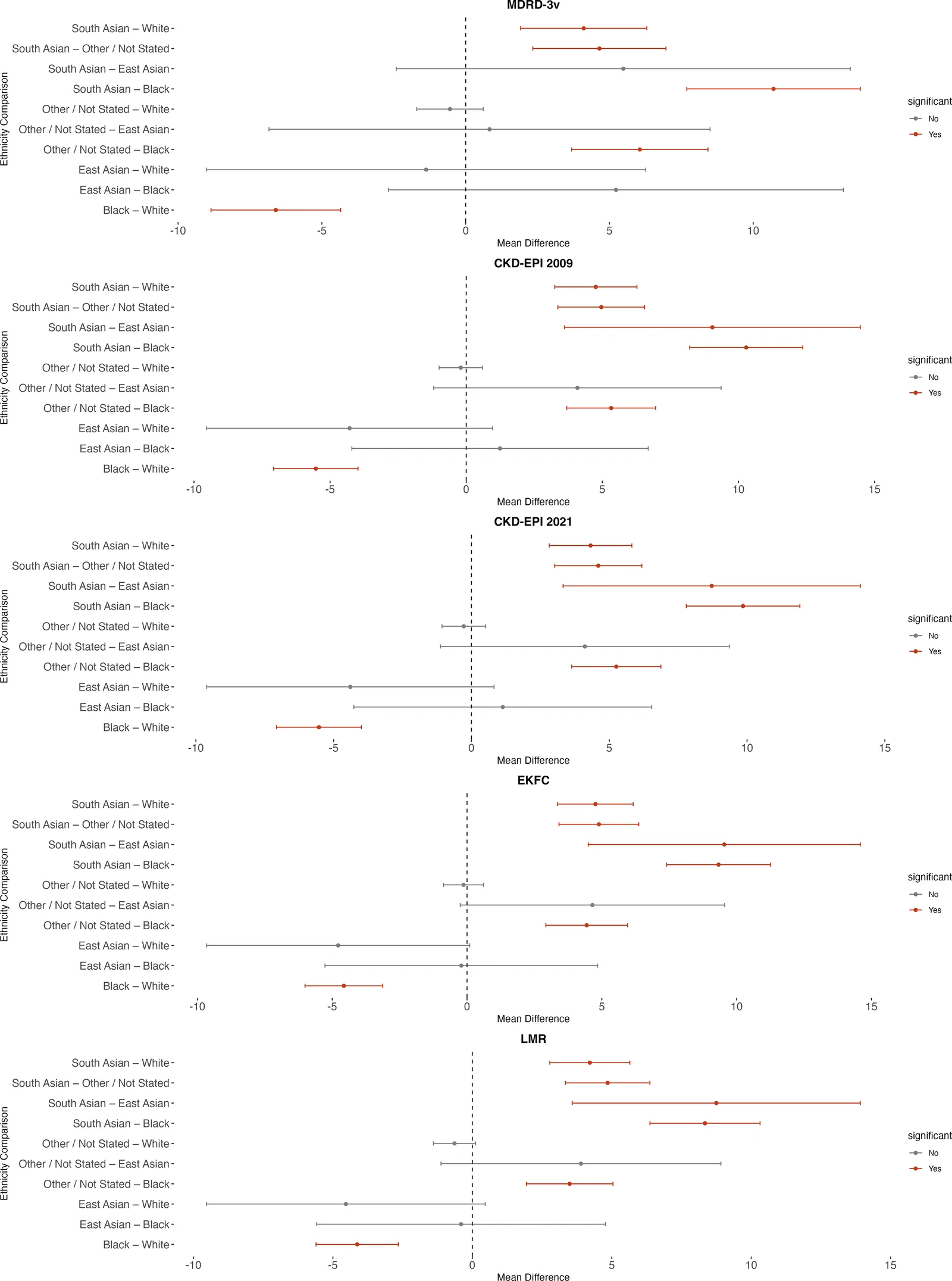
Forest plot showing mean deviation in difference for each ethnicity group comparison, stratified by eGFR equation.

**Fig. 3:**
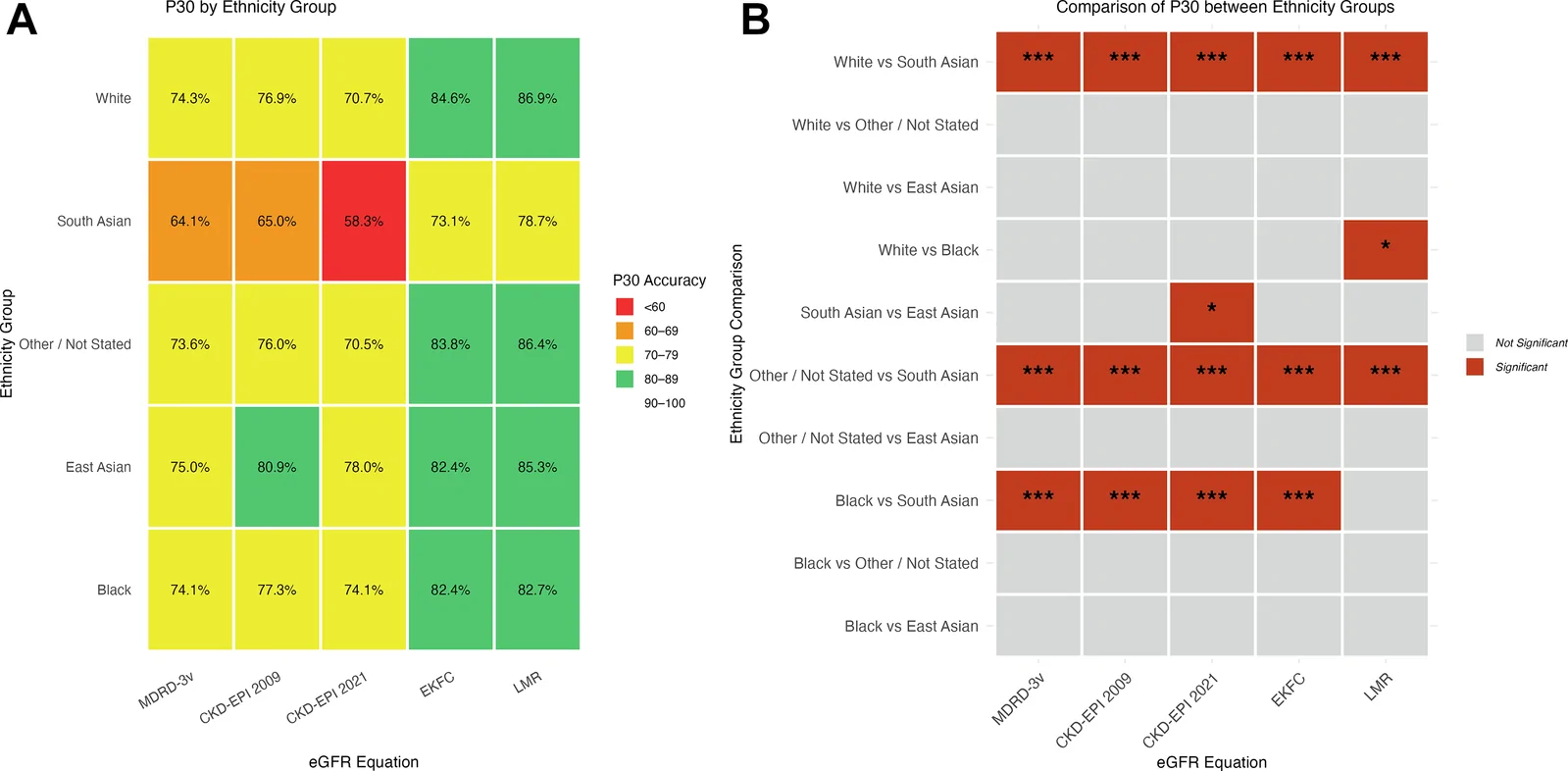
Heatmap identifying (A) accuracy of each eGFR equation stratified by ethnicity group and (B)statistically significant differences in P30 between ethnicity groups. Abbreviations: eGFR = Estimated Glomerular Filtration Rate. P30 thresholds according to KDIGO 2024 CKD guidance: 90–100% = Optimal; 80–89% = Good; <80% = Sub-optimal. ∗p < 0.05; ∗∗ p < 0.01; ∗∗∗ p < 0.001.

**Fig. 4:**
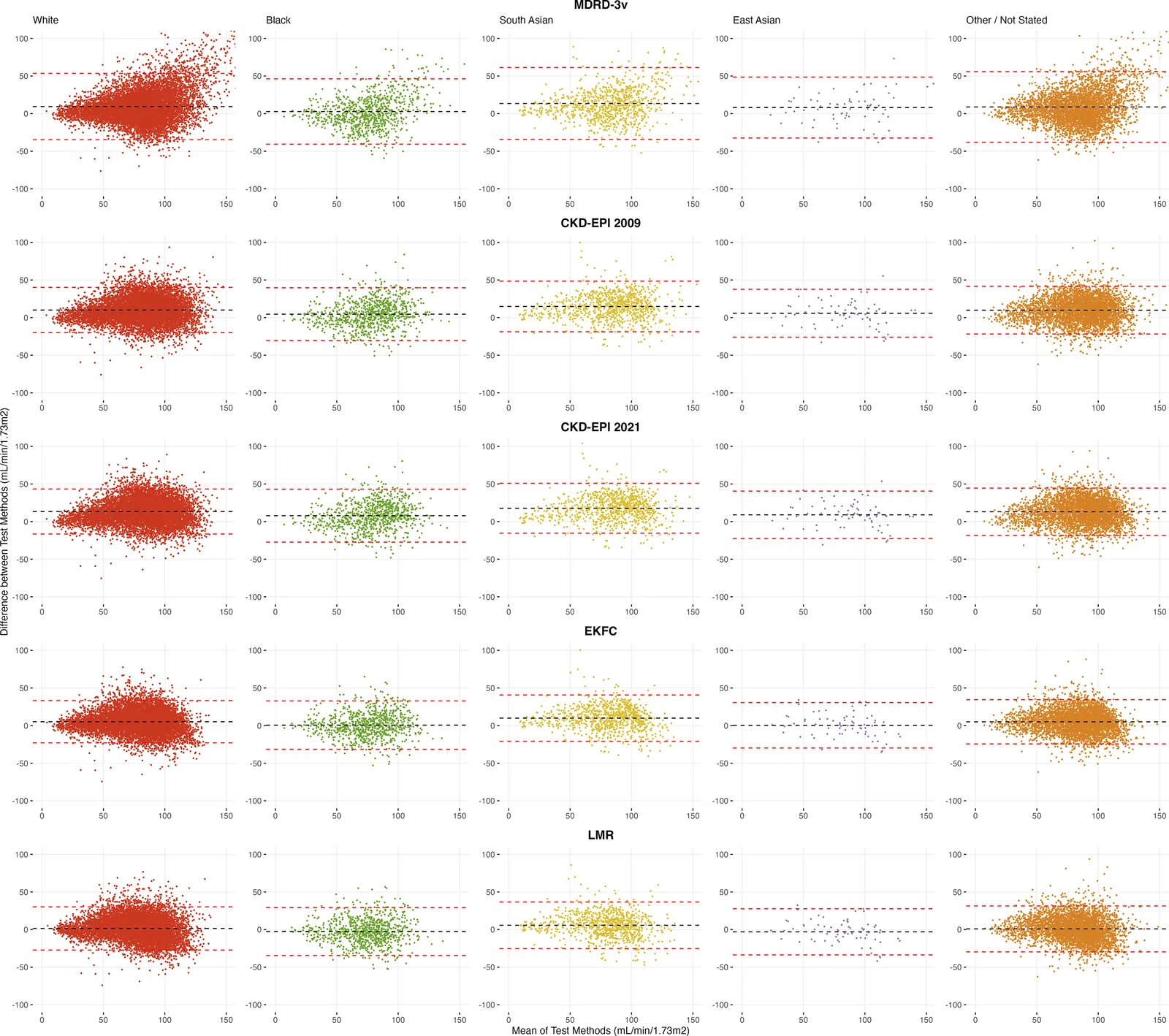
Bland Altman plots demonstrating agreement between eGFR and mGFR for each ethnicity group stratified by eGFR equation.

Overall, difference was higher amongst South Asians compared to other ethnicity groups (Fig. 2). Accuracy was comparable between White, Black, East Asian and Other/Not Stated groups but South Asians had significantly lower accuracy compared to White and Black ethnicity groups (Fig. 3). Limits of agreement remained wide for each ethnicity group but were widest for South Asians (Fig. 4). Difference, precision and P30 for each eGFR equation stratified by ethnicity are illustrated in Supplementary Figure S3.

In participants from White ethnic backgrounds, all eGFR equations overestimated mGFR. LMR (1.5 mL/min/1.73 m^2^; 95% CI: 1.2–1.8 mL/min/1.73 m^2^) and EKFC (5.2 mL/min/1.73 m^2^; 95% CI: 4.9–5.4 mL/min/1.73 m^2^ respectively) had least mean difference, better precision (SD = 14.6 and 14.3 mL/min/1.73 m^2^ respectively) and greater accuracy with P30 values of 86.9% (95% CI: 86.2–87.5%; LMR) and 84.6% (95% CI: 83.8%–85.3%; EKFC). This was compared to the USA derived equations with observed accuracy ranging from 70.7% (95% CI: 69.8%–71.6%) with CKD-EPI 2021 to 76.9% (95% CI: 76.0%–77.7%) with CKD-EPI 2009.

All equations had lower difference in the Black ethnicity group than White, South Asian and Other/Not Stated ethnicity groups. EKFC and MDRD-3v had least difference (0.6 and 2.8 mL/min/1.73 m^2^ respectively) and were not significantly different. EKFC and LMR had the highest accuracy (82.4% and 82.7% respectively), whereas the MDRD-3v and CKD-EPI equations all had P30 values below the 80% KDIGO threshold (Table 2). Compared to the CKD-EPI_2021_, CKD-EPI_2009_ had significantly lower difference (4.5 mL/min/1.73 m^2^; 95% CI: 3.3–5.7 mL/min/1.73 m^2^ vs. 7.9 mL/min/1.73 m^2^, 95% CI: 6.7–9.1 mL/min/1.73 m^2^; p < 0.001) and higher accuracy (P_30_ 77.3%, 95% CI: 74.4–80.1% vs. 74.1%, 95% CI: 71.1–77.0%). eGFR equations including the Black ethnicity coefficient are reported in Supplementary Table S7.

In South Asians, difference was significantly higher for each eGFR equation when compared to other ethnicity groups (p < 0.001). LMR, followed by EKFC had least difference compared to mGFR at 5.7 (95% CI: 4.7–6.8) and 9.9 (95% CI: 8.9–11.0) mL/min/1.73 m^2^ respectively. CKD-EPI_2021_ had greatest difference at 17.7 (95% CI: 16.6–18.8) mL/min/1.73 m^2^ and lowest accuracy (58.3%; 95% CI: 55.1–61.6%). Accuracy was below 80% for all equations (Fig. 3).

### Performance by referral speciality

Baseline characteristics differed by referral speciality (Supplementary Table S8). Participants referred from nephrology were younger than those referred from haematology/oncology and hepatology, with mean age 47.2 ± 13.7 years compared with 55.4 ± 16.0 years and 60.4 ± 14.2 years, respectively (p < 0.001). Mean corrected mGFR was also higher in nephrology referrals than in haematology/oncology and hepatology referrals: 80.4 ± 23.8 mL/min/1.73 m^2^ versus 77.0 ± 23.8 and 60.8 ± 25.7 mL/min/1.73 m^2^, respectively (p < 0.001). Median BMI was higher in nephrology referrals than haematology/oncology and hepatology referrals: 26.7 kg/m^2^ (IQR 23.9–30.2) versus 26.1 kg/m^2^ (23.0–29.8) and 24.4 kg/m^2^ (21.5–28.4), respectively (p < 0.001).

**Fig. 5:**
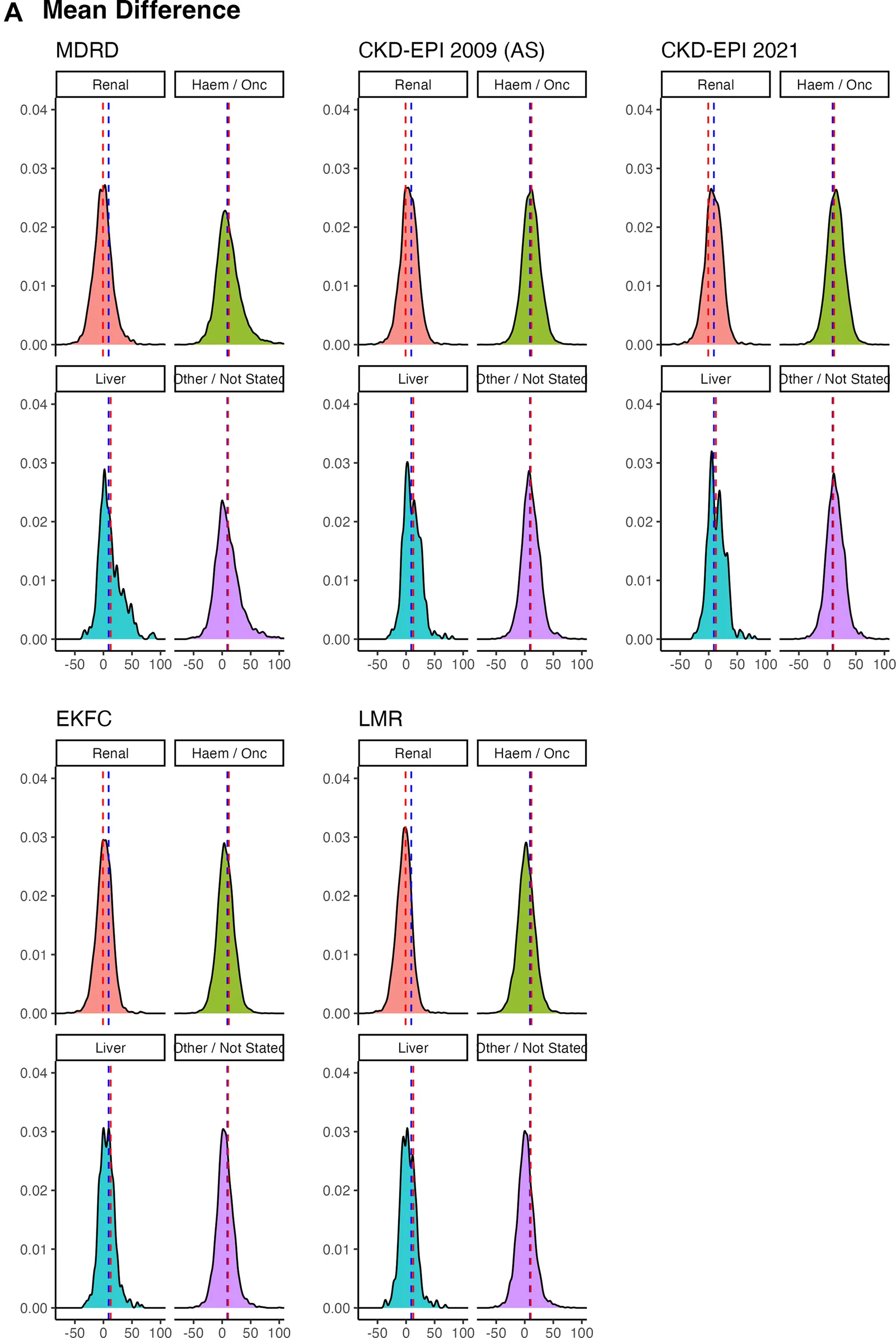

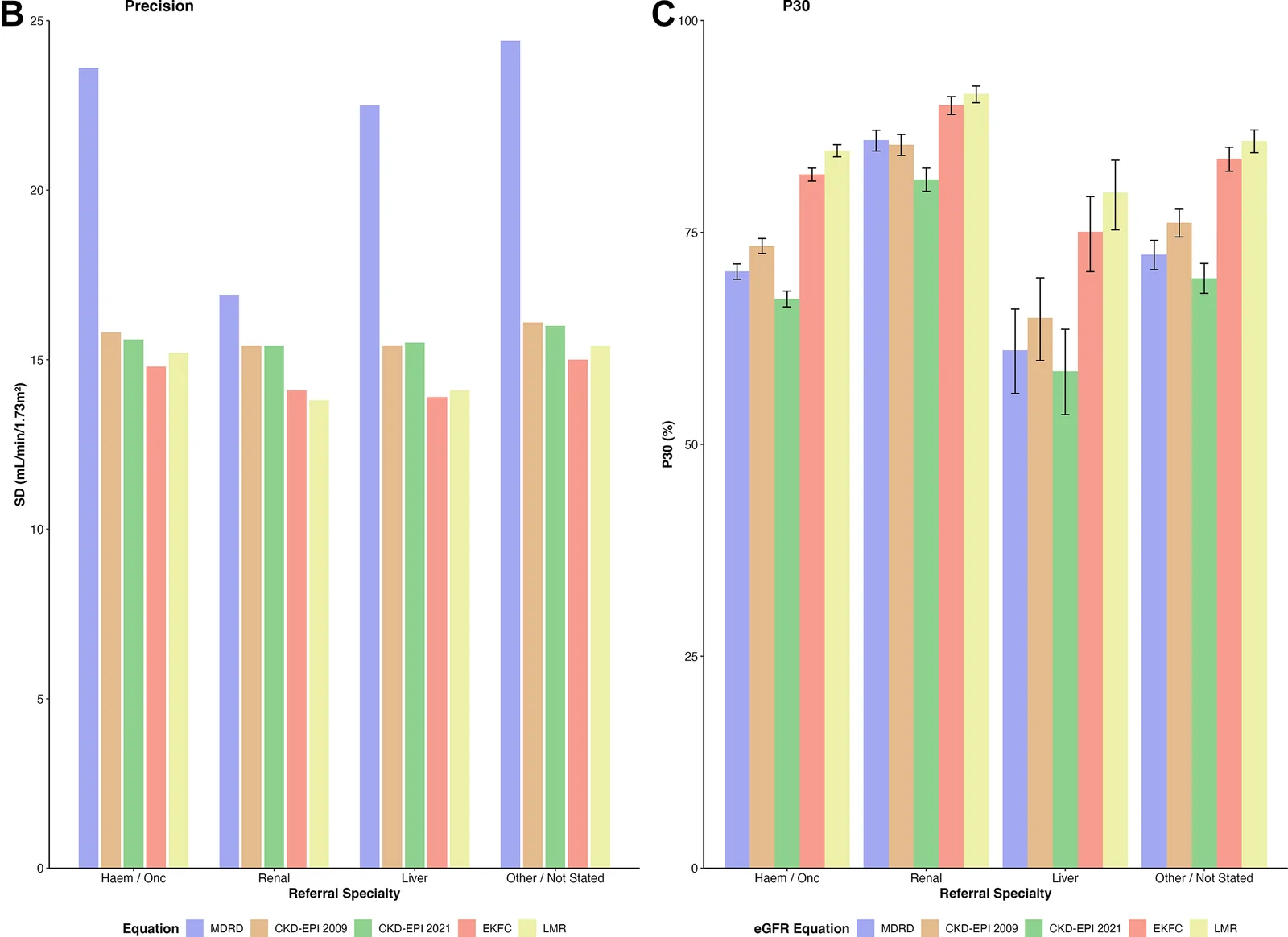
Trends of (A) Mean difference, (B) Precision and (C) P30 of each eGFR equation when stratified by referral teams. (A) Mean Difference is displayed as faceted density plots for each eGFR equation and stratified for referral speciality. The red dashed line represents the overall mean difference for the subgroup. The blue dashed line represents the overall mean difference for the whole cohort. (B) Standard deviation of the mean difference (precision) plotted against eGFR equation is illustrated as a bar chart with stratification for referral speciality. (C) P30 is plotted against eGFR equation and illustrated as a bar chart with stratification for referral speciality.

Performance of eGFR equations differed depending on referral speciality (Fig. 5). Participants referred via nephrology services (n = 3116) had better agreement than other referral specialities (Table 3). LMR and MDRD both demonstrated negative systematic differences (−3.4 and −0.8 mL/min/1.73 m^2^ respectively), whilst the CKD-EPI_2009_ and CKD-EPI_2021_ had the highest positive difference (5.2–8.4 mL/min/1.73 m^2^ respectively). P30 was also substantially greater than other referral specialities ranging from 81.3% (CKD-EPI_2021_) to 91.3% (LMR).

**Table 3:** Difference, precision and accuracy (P30) of the eGFR equations compared to mGFR with stratification by referral speciality.

eGFR Equation	Mean difference, mL/min/1.73 m^2^ [95% CI]	SD	Median difference, mL/min/1.73 m^2^ [95% CI]	IQR, mL/min/1.73 m^2^	RMSE, mL/min/1.73 m^2^	P30, % [95% CI]	95% LoA (lower to upper)
Nephrology (n = 3116)							
MDRD-3v	−0.8 [−1.4 to −0.2]	16.9	−1.1 [−1.8 to −0.5]	−11.2 to 8.6	17.0	85.9 [84.7–87.1]	−34.0 to 32.4
CKD-EPI_2009_	5.2 [4.7–5.8]	15.4	5.1 [4.5–5.8]	−4.2 to 15.2	16.2	85.4 [84.1–86.6]	−24.9 to 35.3
CKD-EPI_2021_	8.4 [7.9–9.0]	15.4	8.5 [7.8–9.2]	−1.2 to 18.7	17.5	81.3 [79.9–82.6]	−21.7 to 38.6
EKFC	1.7 [1.2–2.2]	14.1	2.1 [1.3–2.6]	−6.9 to 10.9	14.2	90.0 [89.0–91.1]	−26.0 to 29.4
LMR	−3.4 [−3.9 to −3.0]	13.8	−2.9 [−3.5 to −2.4]	−11.9 to 5.2	14.2	91.3 [90.4–92.3]	−30.4 to 23.5
Haematology & Oncology (n = 9799)							
MDRD-3v	11.8 [11.4–12.3]	23.6	8.8 [8.3–9.2]	−2.5 to 22.5	26.4	70.4 [69.5–71.3]	−34.35 to 58.01
CKD-EPI_2009_	11.4 [11.1–11.7]	15.8	11.1 [10.7–11.4]	1.1–21.3	19.5	73.4 [72.5–74.3]	−19.59 to 42.31
CKD-EPI_2021_	14.7 [14.4–15.0]	15.6	14.6 [14.2–15.0]	4.5–24.7	21.4	67.2 [66.2–68.1]	−15.90 to 45.22
EKFC	6.1 [5.8–6.4]	14.8	5.8 [5.4–6.1]	−3.2 to 15.5	16.0	81.8 [81.1–82.6]	−22.91 to 35.15
LMR	2.6 [2.3–2.9]	15.2	2.5 [2.1–2.8]	−7.0 to 12.1	15.4	84.7 [84.0–85.4]	−27.17 to 32.37
Hepatology (n = 365)							
MDRD-3v	12.7 [10.4–15.1]	22.5	8.1 [5.3–9.8]	−1.7 to 23.7	25.9	61.1 [56.1–66.1]	−31.4 to 56.9
CKD-EPI_2009_	10.4 [8.9–12.0]	15.4	9.0 [6.3–11.4]	−0.4 to 19.7	18.6	64.9 [60.0–69.8]	−19.8 to 40.7
CKD-EPI_2021_	13.6 [12.0–15.2]	15.5	12.0 [9.1–15.1]	3.0–23.4	20.6	58.6 [53.6–63.7]	−16.8 to 44.1
EKFC	6.2 [4.8–7.7]	14.0	5.8 [3.5–7.7]	−2.9 to 14.3	15.3	75.1 [70.6–79.5]	−21.1 to 33.6
LMR	3.8 [2.4–5.3]	14.1	2.6 [1.5–4.9]	−5.7 to 12.6	14.6	79.7 [75.6–83.9]	−23.8 to 31.4
Other or Not Stated (n = 2599)							
MDRD-3v	10.4 [9.5–11.3]	24.4	6.7 [5.6–7.6]	−4.3 to 20.8	26.5	72.4 [70.7–74.1]	−37.4 to 58.2
CKD-EPI_2009_	10.3 [9.7–11.0]	16.1	9.6 [8.8–10.1]	0.2–20.0	19.1	76.1 [74.5–77.8]	−21.2 to 41.8
CKD-EPI_2021_	13.7 [13.1–14.3]	16.0	13.0 [12.3–13.7]	3.6–23.5	21.0	69.6 [67.8–71.4]	−17.6 to 45.0
EKFC	5.4 [4.8–6.0]	15.0	4.8 [4.0–5.2]	−3.7 to 14.1	15.9	83.7 [82.3–85.1]	−23.9 to 34.7
LMR	2.0 [1.5–2.6]	15.4	1.4 [0.7–2.0]	−7.5 to 10.8	15.5	85.8 [84.5–87.1]	−28.1 to 32.2

In haematology and oncology referrals (n = 9799), all equations overestimated mGFR on average. LMR again showed the smallest mean difference, 2.6 mL/min/1.73 m^2^ (95% CI: 2.3–2.9), and the highest P30 at 84.7% (95% CI: 84.0–85.4). EKFC had the next best performance, with mean difference 6.1 mL/min/1.73 m^2^ (95% CI: 5.8–6.4) and P30 81.8% (95% CI: 81.1–82.6). MDRD-3v, CKD-EPI 2009 and CKD-EPI 2021 had larger positive bias and lower P30, with CKD-EPI 2021 showing the greatest mean difference (Fig. 5A–C).

Hepatology and other or not stated specialities are summarised in Table 3. Concordance correlation of each eGFR equation stratified by referral speciality is illustrated in Supplementary Figure S4.

### Post hoc power analysis

A post hoc power analysis was performed across all five eGFR equations and ethnicity groups. The observed effect size for bias between ethnicity groups was consistent with the original sample size assumption, with Cohen's f ranging from 0.08 to 0.11.

For bias, four ethnicity groups were adequately powered to detect a clinically meaningful difference of 5 mL/min/1.73 m^2^ (power >0.99). However, the East Asian subgroup was underpowered (power = 0.64), with a minimum sample size of 97 participants required to achieve 80% power. For P30, the study was adequately powered to detect a 5% difference in P30 between ethnicity groups across all five equations (power = 1.00).

### Multivariable linear regression

After adjusting for demographic, clinical and analytical covariates, ethnicity remained independently associated with eGFR bias across all five equations (Table 4). Adjusted R^2^ values ranged from 0.09 (EKFC) to 0.19 (MDRD-3v). Compared to White patients, Black and East Asian patients had significantly lower eGFR bias across all equations, with the largest differences observed with MDRD-3v (Black: −9.0 mL/min/1.73 m^2^; 95% CI: −10.6 to −7.5 mL/min/1.73 m^2^) and East Asians (−10.7 mL/min/1.73 m^2^; 95% CI: −16.1 to −5.2 mL/min/1.73 m^2^). South Asian patients had significantly higher eGFR bias across all equations (+4.1 to +4.4 mL/min/1.73 m^2^), compared to White ethnic groups. No significant association was observed for the Other/Not Stated group. Assumptions for linear regressions for each eGFR bias model are illustrated in Supplementary Figure S5.

**Table 4:** Multivariable linear regression reporting the association between ethnicity and eGFR bias (eGFR minus mGFR), adjusted for age, sex, BMI, referral speciality, serum albumin, time between eGFR and mGFR tests, creatinine assay and mGFR filtration marker.

Ethnicity vs White	MDRD-3V	CKD-EPI_2009_	CKD-EPI_2021_	EKFC	LMR
R-squared	0.19	0.11	0.10	0.09	0.14
Black	−9.0 (−10.6 to −7.5)∗∗∗	−6.7 (−7.8 to −5.5) ∗∗∗	−6.6 (−7.7 to −5.5) ∗∗∗	−5.5 (−6.5 to −4.4) ∗∗∗	−5.5 (−6.5 to −4.4) ∗∗∗
East Asian	−10.7 (−16.1 to −5.2) ∗∗∗	−8.4 (−12.2 to −4.5) ∗∗∗	−8.2 (−12.1 to −4.3) ∗∗∗	−7.7 (−11.4 to −4.1) ∗∗∗	−8.3 (−12.0 to −4.7) ∗∗∗
South Asian	+4.2 (2.6–5.9) ∗∗∗	+4.3 (3.1–5.4) ∗∗∗	+4.1 (3.0–5.3) ∗∗∗	+4.4 (3.3–5.5) ∗∗∗	+4.4 (3.3–5.5) ∗∗∗
Other/Not stated	−0.2 (−1.0 to 0.7)	−0.3 (−0.9 to 0.4)	−0.3 (−0.9 to 0.4)	−0.2 (−0.8 to 0.4)	−0.2 (−0.8 to 0.3)

Significance: ∗ p < 0.05; ∗∗ p < 0.01; ∗∗∗ p < 0.001.

Estimates are mean difference in bias (mL/min/1.73 m^2^) with 95% CI.

Reference groups: Ethnicity = White; Sex = Female; Referral Speciality = Nephrology; time between tests = Same Day; Creatinine assay = Enzymatic; mGFR filtration marker = 99m-Tc-DTPA.

### Secondary outcomes: age, sex, BMI and serum creatinine

**Fig. 6:**
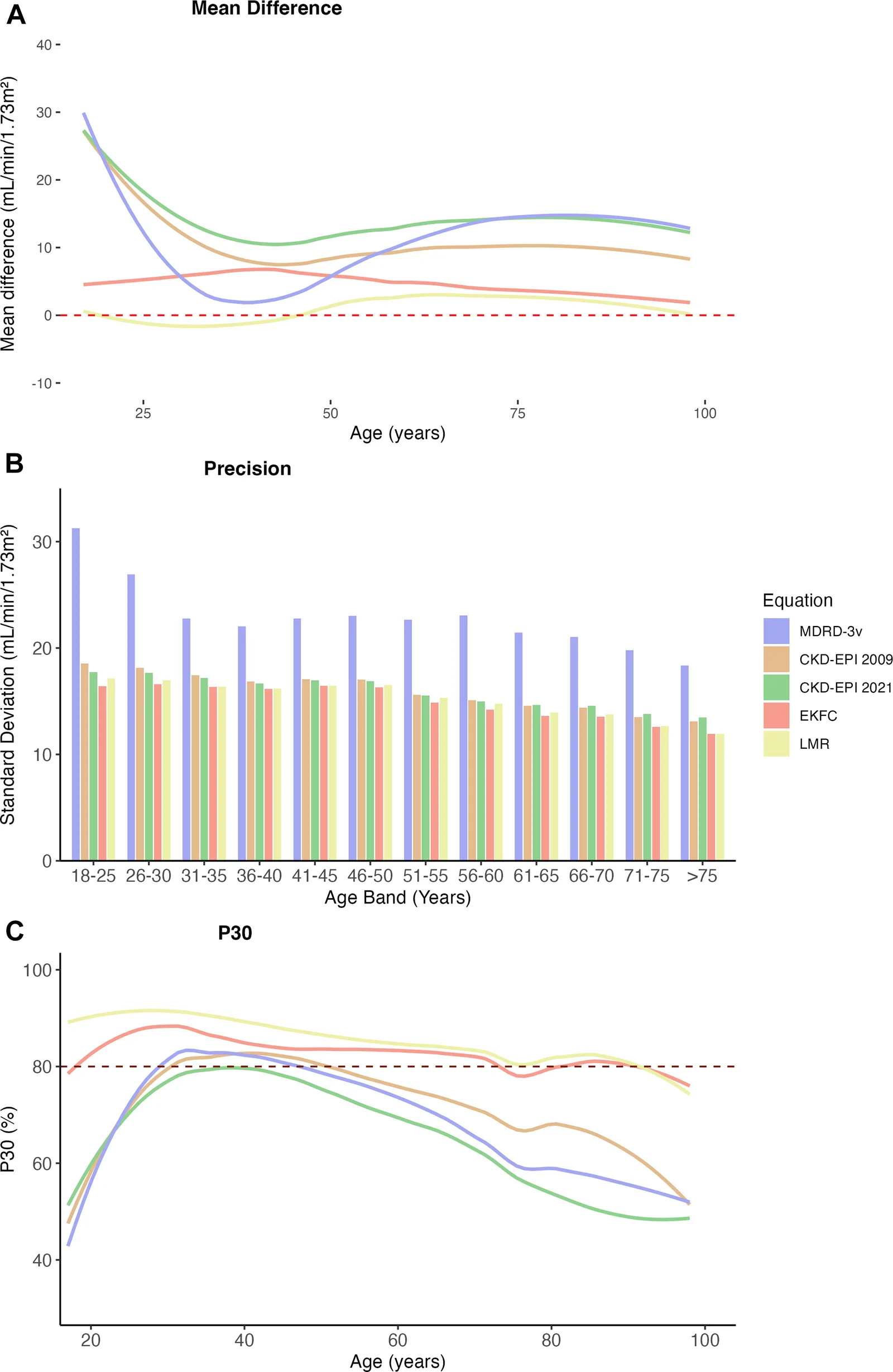
Trends of (A) Mean difference, (B) Precision and (C) P30 of each eGFR equation when stratified by age. (A) Mean difference plotted against age in years as a continuous variable using a LOESS line of best fit. (B) Standard deviation (precision) is plotted as a bar chart against predefined age bands for each eGFR equation. (C) P30 (accuracy) is plotted against age as a continuous variables using a LOESS line of best fit for each eGFR equation. The 80% KDIGO threshold for acceptable P30 is illustrated with the dashed red line.

The EKFC and LMR equations consistently maintained superior P30 accuracy across all ages (Fig. 6). In younger adults (18–25 years), MDRD-3v and CKD-EPI equations had higher positive difference and lower accuracy, compared to EKFC and LMR equations.

All equations reached peak performance in 40–45-year-olds, with declining accuracy with advancing age (e.g. CKD-EPI_2021_ P30: 79.8% vs. 53.0% in 41–45 vs. >75-year-olds respectively) which was less pronounced with the EKFC and LMR equations.

Female sex was associated with greater difference and lower accuracy in all eGFR equations compared to males; however, LMR and EKFC had lowest difference and accuracy greater than 80% in both male and females (Supplementary Figure S6).

Low BMI (<20 kg/m^2^) and low serum creatinine concentrations (<50 μmol/L) were associated with substantial overestimation of mGFR, with reduced precision and poor accuracy for all eGFR equations. With increasing BMI, positive difference was reduced and accuracy improved to over 70% (Supplementary Figure S7). The best performing equations were EKFC and LMR. CKD-EPI_2021_ had lowest accuracy within BMI range of 20–30 kg/m^2^. Similar trends were seen with rising serum albumin concentrations (Supplementary Figure S8) and serum creatinine concentrations (Supplementary Figure S9).

### Misclassification of CKD staging

LMR (65.0%, 95% CI: 64.2–65.7) and EKFC (64.9%, 95% CI: 64.1–65.6) had highest CKD stage classification accuracy compared with mGFR categorised KDIGO CKD stages. CKD-EPI_2021_ performed less well, with only 55.4% (95% CI: 54.6–56.1) correctly classified. The proportion of people correctly classified with eGFR equations decreased with advancing CKD stage (Table 5).

**Table 5:** Proportion of correctly classified CKD stages based on mGFR with stratification by CKD stages 1–5.

	MDRD-3v	CKD-EPI_2009_	CKD-EPI_2021_	EKFC	LMR
Whole Cohort (n = 15,879)	60.1 (59.3–60.9)	59.6 (58.9–60.4)	55.4 (54.6–56.1)	64.9 (64.1–65.6)	65.0 (64.2–65.7)
CKD Stage 1 (n = 5100)	72.6 (71.3–73.8)	86.9 (86.0–87.8)	91.4 (90.6–92.1)	78.5 (77.3–79.6)	63.9 (62.6–65.2)
CKD Stage 2 (n = 7131)	54.8 (53.6–55.9)	45.8 (44.6–46.9)	36.3 (35.2–37.4)	60.0 (58.9–61.1)	70.4 (69.3–71.4)
CKD Stage 3 (n = 3105)	54.1 (52.4–55.9)	49.1 (47.3–50.8)	42.7 (40.9–44.4)	56.7 (55.0–58.4)	55.6 (53.8–57.3)
CKD Stage 4 (n = 476)	48.1 (43.7–52.6)	45.8 (41.4–50.3)	40.3 (36.0–44.8)	49.0 (44.5–53.4)	60.3 (55.8–64.6)
CKD Stage 5 (n = 67)	41.8 (30.7–53.7)	41.8 (30.7–53.7)	38.8 (28.1–50.8)	43.2 (32.1–55.2)	41.8 (30.7–53.7)

CKD = chronic kidney disease.

Proportion of correctly classified participants stratified by eGFR equation and CKD stage is reported with 95% confidence intervals.

**Fig. 7:**
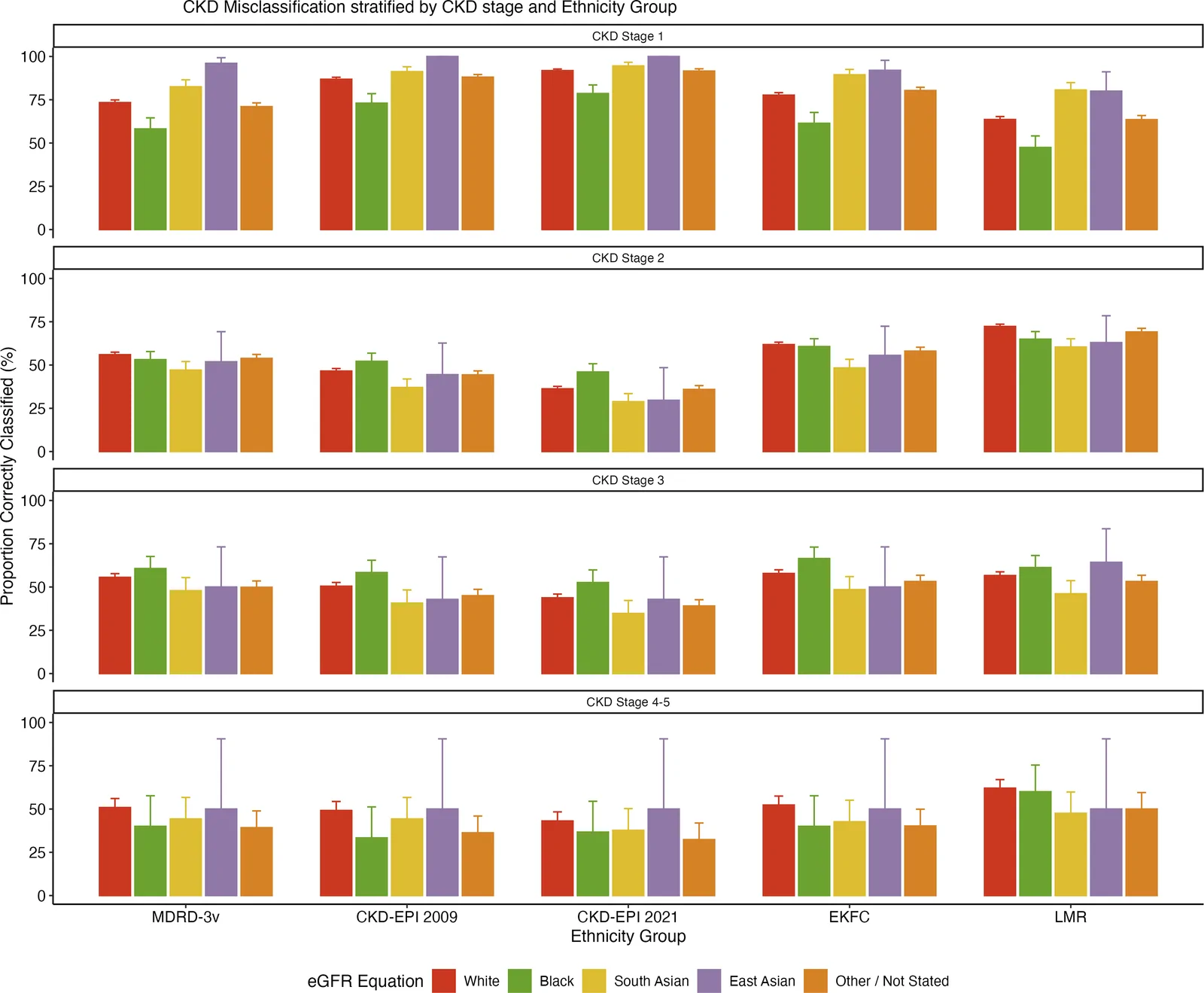
Bar Chart showing proportion of correctly classified mGFR-defined CKD stages, stratified by CKD stage and faceted by ethnicity. Error bars represent 95% confidence intervals.

When stratified by ethnicity the proportion of participants with correctly classified CKD staging was consistently higher with EKFC and LMR equations. Misclassification was highest for South Asians (Fig. 7) particularly with the CKD-EPI_2021_ equation which correctly classified only 50.7% of people. When stratifying by referral speciality, participants referred by Renal services had higher proportions correctly classified compared to those referred by Haematology/Oncology or Hepatology services (Supplementary Table 9).

### Potential impact on prescribing practices

A substantial proportion of patients would have been classified as ineligible based on eGFR alone, due to eGFR not meeting nationally recommended prescribing thresholds. For example, with dapagliflozin is licenced for treatment of proteinuric kidney disease with or without diabetes mellitus with initiation occurring between an eGFR of 25–75 mL/min/1.73 m^2^. There were 6586 participants who had an mGFR result within these thresholds. The proportion of participants with potential prescribing ineligibility based on eGFR alone of dapagliflozin based on their eGFR result, ranged from 29.4% (LMR) to 52.1% (CKD-EPI_2021_). A similar trend was seen for each medication analysis with LMR showing the smallest proportion of potentially missed opportunities if used to guide prescribing.

Only small proportions of participants might have prolonged prescribing eligibility based on eGFR alone at mGFR levels below the target threshold ranging from 0.4% (CKD-EPI_2021_) for ramipril or metformin to 7.2% (LMR) for empagliflozin (Table 6).

**Table 6:** Number of participants in the overall cohort, who would fall within the treatment ranges (for initiation, cessation and dose adjustment) based on their mGFR result.

Drug name (therapeutic mGFR range, mL/min/1.73 m^2^)	mGFR within range, N	MDRD-3v		CKD-EPI_2009_		CKD-EPI_2021_		EKFC		LMR	
		Below	Above	Below	Above	Below	Above	Below	Above	Below	Above
Dapagliflozin (25–75)	6586	69 (1.0)	2469 (37.5)	60 (0.9)	2980 (45.2)	48 (0.7)	3433 (52.1)	60 (0.9)	2393 (36.3)	111 (1.7)	1933 (29.4)
Empagliflozin (25–45)	1438	63 (4.4)	696 (48.4)	54 (3.8)	744 (51.7)	42 (2.9)	858 (59.7)	54 (3.8)	683 (47.5)	103 (7.2)	646 (44.9)
Empagliflozin (45–90)	9326	239 (2.6)	3048 (32.7)	182 (2.0)	3960 (42.5)	120 (1.3)	4865 (52.2)	218 (2.3)	2688 (28.8)	264 (2.8)	1815 (19.5)
Ramipril/Metformin (≥30)	15,336	98 (0.6)	–	94 (0.6)	–	69 (0.4)	–	94 (0.6)	–	173 (1.1)	–
Fineronone (25–60)	3476	66 (1.9)	1484 (42.7)	57 (1.6)	1656 (47.6)	45 (1.3)	1892 (54.4)	57 (1.6)	1405 (40.4)	108 (3.1)	1361 (39.2)

The number and percentage proportion of participants who would be inappropriately managed for these treatments is reported for each equation, divided into below and above the target threshold.

When stratified by ethnicity, the proportion of patients with eGFR results above drug initiation thresholds was consistently higher in South Asian and ‘Other/Not Stated’ groups (Supplementary Table S10). LMR performed best and CKD-EPI_2021_ performed worst across all ethnicity groups.

## Discussion

Following removal of ethnicity coefficients from eGFR equations, both national and international bodies have recommended re-evaluating use of eGFR equations at regional levels to determine optimal performance. Our study is the second largest of adults with concurrent mGFR and creatinine-based eGFR testing and includes the greatest number of individuals from South Asian backgrounds. All five creatinine-based eGFR equations systematically overestimated mGFR, with worse performance in South Asian populations. LMR and EKFC equations consistently demonstrated superior performance across all agreement metrics including difference, precision, 30% accuracy (P30) and limits of agreement, compared to the CKD-EPI_2009_, CKD-EPI_2021_ and MDRD-3v equations. The CKD-EPI_2021_ equation performed poorly in both White and non-White populations with accuracy falling below the 80% threshold recommended by KDIGO for clinical decision making across all ethnicity groups.

South Asians had poorest agreement between eGFR and mGFR characterised by greater difference and lower 30% accuracy (P30). In all cases, P30 was less than the 80% benchmark set by The KDIGO CKD Guidelines in 2024 for clinical decision-making. This finding has been demonstrated in similar studies evaluating eGFR performance in South Asian cohorts, which suggests that creatinine-based eGFR is suboptimal in this group. This is concerning, given the increased risk of rapid kidney disease progression in South Asians and other ethnic minorities,^16^ these misclassifications of CKD diagnosis and severity, which underestimate kidney failure risk, delay transplant listing and impact prescribing opportunities for early preventative interventions could contribute to healthcare inequity within this population.

The reason for the poor performance of creatinine-based eGFR is difficult to explain within the limits of our dataset. South Asian populations may have differing characteristics which impact non-GFR determinants of creatinine, including body composition, diet and genetic ancestry when compared to White populations. Emerging data from Northern India also suggests that the median serum creatinine concentration and median mGFR in a healthy Northern Indian population is lower than the European reference values, which are used in the EKFC equation calibration.^17^ This would contribute to systematic bias seen in South Asians, however, these findings require replication and validation and therefore it remains unclear if this trend would be present within the wider South Asian diaspora.

The LMR and EKFC equations, despite being derived using iohexol-based mGFR measurements in White European populations, demonstrated smaller difference and higher accuracy across ethnic groups and other subgroups, including age categories, CKD stages, and referral specialities when compared to MDRD-3v and CKD-EPI equations. Importantly, performance of both equations in our cohort was comparable to that reported in derivation studies.^10^^,^^14^ The EKFC equation, which is validated from ages over 2-years old and incorporates a rescaled creatinine constant (Q-value) that can be tailored to local populations shows promise, particularly as it has recently been recommended as the first-line eGFR equation by the European Federation of Clinical Chemistry and Laboratory Medicine.^18^

Interestingly, the LMR performed better in ethnic minority populations including South Asians (although this remained suboptimal), despite these equations being developed in a White Swedish cohort. This finding likely reflects that equation performance is dependent on multiple factors, which include equation structure, calibration, age and sex handling, GFR distribution, clinical setting and non-GFR determinants such as body composition, diet and genetic ancestry. In our cohort, this may be illustrated in younger adults, where young age was associated with overestimation of mGFR with reduced accuracy by eGFR equations except for LMR and EKFC, likely reflecting better modelling of age in LMR and EKFC equations.

An important trade-off with LMR and to a lesser extent EKFC was observed in the CKD stage classification. For individuals with mGFR ≥90 mL/min/1.73 m^2^, LMR correctly classified a lower proportion of individuals compared to MDRD-3v or the CKD-EPI equations. This likely reflects the tendency of LMR to generate lower eGFR estimates compared to other equations. Although this may reduce systematic overestimation in some settings, it may also increase a shift in CKD stage from G1 to G2 in individuals with preserved mGFR. This may negatively impact CKD diagnosis and coding, surveillance intensity, medication thresholds and prevalence estimates and needs to be weighed against the better overall agreement observed with LMR.

Low BMI, low serum creatinine and referral from haematology/oncology and hepatology services were also associated with overestimation of mGFR by eGFR equations, likely related to reduction in muscle mass, a well-known limitation of creatinine-based eGFR.^19^

This is particularly relevant to our cohort as haematology and oncology referrals for mGFR studies represented 61.7% of the population. In contrast 19.6% were referred from nephrology, where the likely indications were prospective kidney donor evaluation or concerns regarding creatinine-based eGFR. This is supported by our results, in which patients referred by nephrology services have smaller mean differences in eGFR from mGFR and P30 greater than 80% for all equations, compared to haematology and oncology referrals which ranged from 67.2% to 84.7%. Differences in muscle mass and diet may both have had an impact on creatinine generation without affecting mGFR, which would inflate the systematic overestimation seen in our cohort. This is weakly supported by haematology and oncology patients having a significantly lower BMI compared to nephrology patients, although this remains a very poor surrogate for muscle mass.

These findings underscore the limitations of serum creatinine as a GFR biomarker and reinforces the need for alternative biomarkers and individualised interpretation of eGFR.^20^^,^^21^ In addition, this reinforces that the degree of systematic overestimation observed in the whole cohort should not be interpreted as equation performance in an unselected population, but instead reflects performance in clinically selective populations in whom accurate GFR assessment is often required.

Although longitudinal accuracy in detecting change in kidney function is important for monitoring progression of disease and guiding clinical intervention, cross-sectional agreement between eGFR and mGFR remains clinically relevant. Clinical and policy decisions are often threshold-based on single time points, including CKD staging, coding, referral criteria, epidemiological estimates of CKD prevalence, drug dosing and prescribing eligibility.

Our simulated implications of eGFR equation use highlighted differences in potential prescribing eligibility, CKD classification and potential impact on prevalence estimates which provides practical insights for clinicians and policy makers considering implementation of new eGFR equations. For example, using CKD-EPI_2009_ equation could lead to potential prescribing ineligibility based on eGFR alone, of SGLT2i treatment in 52.6% of South Asians compared to 33.7% and 44.6% of Black and White participants respectively. This finding needs to be balanced against the premise that GFR thresholds for medications such as dapagliflozin are based on eGFR rather than mGFR and therefore it remains unclear as to whether there would be real-world benefit or harm based on a change in eGFR equation. However, because prescribing guidance and service eligibility are commonly implemented using eGFR thresholds, equation-dependent differences in eligibility classification remain relevant to equity, access and policy implementation.

Strengths of our study include that we evaluated a large ethnically diverse cohort from multiple UK centres encompassing a wide age range. The observational nature of this study allowed for potentially greater representation, particularly from ethnic minority groups, compared to prospective studies, which typically struggle to recruit non-White participants.^22^ Our findings of positive difference with all eGFR equations contrast with those of the recent UK eGFR-C study (n = 1167) which reported negative difference and high accuracy (P30 values ≥ 89.5% for all primary equations), but was underpowered to explore performance according to ethnicity.

The eGFR-C cohort included participants with CKD stage 3 and excluded those with malignancy and other comorbidities, whereas we assessed equation performance in a diagnostically challenging population where highly accurate GFR is clinically mandated (e.g. chemotherapy dosing or transplant evaluation). This emphasises the unreliability of creatinine-based eGFR in these specific patient groups and reinforces the critical role of mGFR in this context. Importantly, each eGFR equation was paired against mGFR within the same individuals, maintaining the validity of comparisons between equations within this cohort.

Ethnicity was captured from electronic health records, which may be self-reported, or observer reported. The latter may introduce the risk of misclassification bias. In addition, 19.0% of participants had no ethnicity stated, which may under-represent ethnic minority groups, as this matches trends in the literature when retrospectively interrogating electronic health records, which likely include observer-reported ethnicity.^23^^,^^24^

To improve the likelihood of achieving adequate numbers for comparison, ethnicity categories were grouped into broad analysis groups. This was necessary for statistical precision but given ethnicity is a heterogenous social construct, this approach is also likely to mask within-group differences. Despite the large cohort size, East Asian participants were underpowered limiting inference for this population. However, this distribution is likely to be reflective of UK populations and clinical context, where census provide greater granularity for bigger population groups. For example, South Asian included Indian, Bangladeshi and Pakistani. These features should be considered when comparing our findings with international eGFR validation studies, particularly those using alternative ethnic categories or cohorts from East Asia.

Limitations include the retrospective design, which may introduce selection bias and use of routine data. Consequently, cystatin-C, an alternative biomarker to creatinine which is not limited by the same non-GFR determinants as creatinine, was not included as it is infrequently and inconsistently utilised in adult populations in the UK. The main indications for formal GFR assessment in the UK are chemotherapy dosing or transplant donor workup,^25^^,^^26^ and results are likely to reflect a comorbid population or those being investigated for unexpected discrepancies between eGFR and anticipated GFR. Extrapolation of findings to average healthy populations and other diseases needs to be made with caution. However, site selection enabled large numbers of ethnic minorities, who may not have been invited or declined to participate in a prospective cohort to be included thus this approach may have enabled wider representation of different socioeconomic, age and cultural groups which has not previously been possible.

Apart from White participants, some ethnicity-specific subgroup analyses were limited by small numbers, particularly within the East Asian cohort and/or individuals with CKD stages 4–5, restricting the interpretation of equation performance in within these subgroup analyses. Furthermore, it was not possible to explore eGFR equation accuracy in people who identify with different genders from sex assigned at birth, as these data were unavailable. These underrepresented groups in research, likely to have differences in muscle mass, diet and creatinine secretion and consequent eGFR equation performance warrant assessment.

There were analytical differences between sites including differences in creatinine assays, mGFR filtration markers, possibly mGFR method protocols and likely variation in referral practice, but all creatinine results were IDMS-traceable, there were no significant differences identified in multivariable regression analysis between assays and only small differences in eGFR estimates between mGFR filtration markers were identified.^27^ In addition, site-level variation in data extraction procedures and the absence of duplicate independent data extraction increase the risk of error. As this was a retrospective analysis of routinely collected NHS clinical data, individual results could not be independently remeasured or recoded for research purposes; residual data limitations may therefore remain because of between-site variation in measurement protocols, coding completeness and extraction pathways. Finally, we were unable to evaluate longitudinal equation performance or accuracy for detecting true change in GFR over time.

In conclusion, our findings add to growing international evidence suggesting that continued reliance on CKD-EPI_2009_ or CKD-EPI_2021_ equations may be suboptimal in some non-US populations, where creatinine-based eGFR is less reliable, and could contribute to health inequalities in CKD care. The observed performance of LMR and EKFC equations across ethnic groups highlights their potential utility in diverse populations and warrants further evaluation in broader clinical settings. Adoption of alternative equations may have implications for public health planning in the UK and beyond, including potential changes in national prevalence estimates, prescribing, and clinical management. Such approaches may also support efforts to improve equity in CKD care for underserved populations disproportionately affected by kidney disease.

## Contributors

RMG and KB conceived the study. RMG, KB, AMP, SG, KD, and PD developed the study methodology. KB was the Chief Investigator. Principal Investigators at participating sites were RMG, DB, SD, DG, JSL, RM, KM, SM, ES, JS, and PC. Investigation and data collection were undertaken by RMG, DB, SC, SD, AD, LE, DG, SG, NH, SK, JSL, TL, NL, RM, KM, TM, SM, JP, AMP, HS, ES, RS, JS, MTo, MTu, AU, and PC. Data curation and cleaning were undertaken by RMG, SK, JSL, TL, NL, RM, KM, TM, JP, HS, RS, JS, MTo, MTu, and AU. RMG prepared the data visualisations and coordinated project administration. RMG and KB wrote the original manuscript draft. RMG, DB, SC, KD, SD, PD, AD, LE, JF, DG, SG, RH, NH, PJ, SK, JSL, TL, NL, RM, KM, TM, SM, JP, AMP, HS, ES, RS, JS, MTo, MTu, AU, PC, and KB critically reviewed, edited and approved the final version of the manuscript.

## Data sharing statement

Data will be made available upon request following publication of this manuscript, subject to consent from local information governance at each site and with an appropriate signed data access agreement in place.

## Declaration of interests

DB and KB have received funding from AstraZeneca and NIHR. PD is consultant for Nephrolyx. SD has received consultancy fees and support for meetings from Takeda, Chiesi, Sandoz, Boehringer Ingelheim, Hansa and funding from NIHR, Kidney Research Yorkshire and Leeds Hospital Charities. PJ has received funding from Boehringer Ingleheim, Novartis and Eli Lilly.

## References

[1] Bikbov B., Purcell C.A., Levey A.S. (2020). Global, regional, and national burden of chronic kidney disease, 1990–2017: a systematic analysis for the global burden of disease study 2017. Lancet.

[2] Couser W.G., Remuzzi G., Mendis S., Tonelli M. (2011). The contribution of chronic kidney disease to the global burden of major noncommunicable diseases. Kidney Int.

[3] Stevens P.E., Ahmed S.B., Carrero J.J. (2024). KDIGO 2024 clinical practice guideline for the evaluation and management of chronic kidney disease. Kidney Int.

[4] Heerspink H.J.L., Stefánsson B.V., Correa-Rotter R. (2020). Dapagliflozin in patients with chronic kidney disease. N Engl J Med.

[5] Levey A.S., Stevens L.A., Schmid C.H. (2009). A new equation to estimate glomerular filtration rate. Ann Intern Med.

[6] Levey A.S., Bosch J.P., Lewis J.B., Greene T., Rogers N., Roth D. (1999). A more accurate method to estimate glomerular filtration rate from serum creatinine: a new prediction equation. Modification of diet in renal disease study group. Ann Intern Med.

[7] Fabian J., Kalyesubula R., Mkandawire J. (2022). Measurement of kidney function in Malawi, South Africa, and Uganda: a multicentre cohort study. Lancet Glob Health.

[8] Yadav A.K., Kaur J., Kaur P. (2024). Evaluation of race-neutral glomerular filtration rate estimating equations in an Indian population. Kidney Int Rep.

[9] Inker L.A., Eneanya N.D., Coresh J. (2021). New Creatinine- and cystatin C–Based equations to estimate GFR without race. N Engl J Med.

[10] Pottel H., Björk J., Courbebaisse M. (2021). Development and validation of a modified full age spectrum creatinine-based equation to estimate glomerular filtration rate. Ann Intern Med.

[11] Zhao L., Li H., Liu H. (2023). Validation of the EKFC equation for glomerular filtration rate estimation and comparison with the Asian-modified CKD-EPI equation in Chinese chronic kidney disease patients in an external study. Ren Fail.

[12] National Institute for Health and Care Excellence (2021). Chronic kidney disease: assessment and management. NICE guideline NG203. https://www.nice.org.uk/guidance/ng203.

[13] NHS England (2021). NHS England National data opt-out open data dashboard. https://digital.nhs.uk/dashboards/national-data-opt-out-open-data.

[14] Nyman U., Grubb A., Larsson A. (2014). The revised Lund-Malmö GFR estimating equation outperforms MDRD and CKD-EPI across GFR, age and BMI intervals in a large Swedish population. Clin Chem Lab Med.

[15] Bland J.M., Altman D.G. (1986). Statistical methods for assessing agreement between two methods of clinical measurement. Lancet.

[16] Mathur R., Dreyer G., Yaqoob M.M., Hull S.A. (2018). Ethnic differences in the progression of chronic kidney disease and risk of death in a UK diabetic population: an observational cohort study. BMJ Open.

[17] Yadav A.K., Kaur J., Kaur R. (2026). Recalibration of the European Kidney Function Consortium eGFR equation for the Indian population. Kidney Int Rep.

[18] Cavalier E., Zima T., Datta P. (2025). Recommendations for European laboratories based on the KDIGO 2024 clinical practice guideline for the evaluation and management of chronic kidney disease. Clin Chem Lab Med.

[19] Nankivell B.J., Nankivell L.F.J., Elder G.J., Gruenewald S.M. (2020). How unmeasured muscle mass affects estimated GFR and diagnostic inaccuracy. EClinicalMedicine.

[20] Titan S.M., Lieske J.C., Meeusen J.W. (2025). Performance of creatinine and cystatin-based equations on estimating measured GFR in people with hematological and solid cancers. Clin J Am Soc Nephrol.

[21] Costa e Silva V.T., Gil L.A., Inker L.A. (2024). Glomerular filtration rate estimation using β2-Microglobulin and β-Trace protein in adults with solid tumors: a prospective cross-sectional study. Am J Kidney Dis.

[22] Routen A., Bodicoat D., Willis A., Treweek S., Paget S., Khunti K. (2022). Tackling the lack of diversity in health research. Br J Gen Pract.

[23] Saunders C.L., Abel G.A., El Turabi A., Ahmed F., Lyratzopoulos G. (2013). Accuracy of routinely recorded ethnic group information compared with self-reported ethnicity: evidence from the English cancer patient experience survey. BMJ Open.

[24] Jones C.P., Truman B.I., Elam-Evans L.D. (2008). Using ‘socially assigned race’ to probe white advantages in health status. Ethn Dis.

[25] Ebert N., Bevc S., Bökenkamp A. (2021). Assessment of kidney function: clinical indications for measured GFR. Clin Kidney J.

[26] van Warmerdam L.J.C., Rodenhuis S., ten Bokkel Huinink W.W., Maes R.A.A., Beijnen J.H. (1995). The use of the Calvert formula to determine the optimal carboplatin dosage. J Cancer Res Clin Oncol.

[27] Balouzet C., Michon-Colin A., Dupont L. (2023). Comparison of 99mTc-DTPA and 51Cr-EDTA for glomerular filtration rate measurement with the continuous infusion method. J Nephrol.

