# Supplementary material for: Performance of estimated glomerular filtration rate equations across ethnic groups in the AIM CKD UK cohort: a multicentre cross-sectional study
Source: Lancet Reg Health Eur. 2026 Aug 25;69:101828. doi: 10.1016/j.lanepe.2026.101828 (PMC13527474; doi:10.1016/j.lanepe.2026.101828)
Supplement: Supplementary file 1 [file mmc1.docx]

**Supplementary Materials**

**Supplementary Methods**

Estimated glomerular filtration rate (eGFR) equation selection rationale

The selection of eGFR equations was chosen to provide a focussed and non-exhaustive comparison of equations which have or have had direct relevance to recent, current and potentially future clinical practice in Europe.

The CKD-EPI 2009 without ethnicity coefficient (CKD-EPI_2009_) equation was chosen because it remains widely used in Europe, internationally and is currently recommended by the National Institute for Health and Care Excellence (NICE) for clinical practice in the UK.^14^ The CKD-EPI 2021 ‘race-neutral’ (CKD-EPI_2021_) equation is the major ‘race-free’ successor to CKD-EPI 2009 and has been adopted in parts of the USA following the recommendations of the National Kidney Foundation – American Society of Nephrology (NKF-ASN) joint task force.^13^ In the UK, this equation was published shortly after the most recent guidelines and therefore there remains questions regarding adoption in the UK. Outside of the United States of America (USA), this equation has been reported to have substantial difference and lower accuracy compared to its predecessor and the European eGFR equations in both Eastern / Southern African and Northern Indian cohorts.^8,9^ Therefore, inclusion of this equation was important to evaluate its performance in a diverse UK cohort.

The MDRD without ethnicity coefficient (MDRD-3v) equation was chosen because it is still used by a substantial minority of UK laboratories in clinical practice, despite not being recommended by NICE since 2014. This was highlighted by an audit published by the UK National External Quality Assessment Service (NEQAS), who reported that 32% of respondent laboratories were using the MDRD equation either with or without the ethnicity coefficient.^28^ We chose to remove the ethnicity coefficient from this equation because this aligned with current clinical recommendations in the UK (although this does not specifically reference the MDRD equation) and also because previous work we completed at a single site in the UK highlighted that using the ethnicity coefficient led to overestimation of mGFR in Black British cohort.

The European Kidney Function Consortium (EKFC) was included due to prior studies in European cohorts, demonstrating strong performance in predominantly White European populations. In the African Research on Kidney Disease (ARK) cohort, the creatinine-based EKFC equation had smaller difference and improved accuracy when compared to the creatinine-based CKD-EPI equations and therefore a further question was would this be replicated in a Black British cohort.^7^ Finally, this equation has subsequently been recommended by the European Federation of Clinical Chemistry and Laboratory Medicine as a European alternative to CKD-EPI warranting its inclusion in this evaluation.^18^

Finally, the fifth equation chosen was the Lund-Malmo Revised (LMR), which was included as it was developed using a large European cohort and has demonstrated comparable or superior performance to CKD-EPI in several European validation studies.^13^ However, data in ethnically diverse groups is lacking, therefore this was a relevant benchmark for assessing equation performance in a diverse UK population.

Cockcroft-Gault was not included in this analysis, despite still being widely used in drug-dosing for certain types of medications, including chemotherapy and direct oral anticoagulants. It was not included because the version of Cockcroft-Gault used in drug formularies is non-indexed for body surface area (BSA) and instead measures creatinine clearance in ml/min. Whereas our study compared BSA-indexed mGFR to BSA-indexed eGFR (both mL/min/1.73m^2^). Evaluation of Cockcroft-Gault would require comparison with absolute mGFR and de-indexed eGFR which was outside the scope of our analysis.

Black ethnicity coefficient

The black ethnicity coefficient was not included in the primary analysis evaluating the performance of eGFR equation in Black British individuals. This is in line with international guidelines which recommend the removal of the Black ethnicity coefficient in clinical practice. We acknowledge there remains evidence suggesting that removal of the Black ethnicity coefficient in US populations may lead to systematic misclassification. However, this is contrasted with substantial evidence highlighting that inclusion of the Black ethnicity coefficient leads to greater systematic overestimation of mGFR in Black individuals in Europe and Africa.^7,29,30^ Therefore, for completeness we have included a sensitivity analysis evaluating agreement of eGFR with mGFR in Black individuals which includes the MDRD and CKD-EPI equations, with their respective ethnicity coefficients (1.16 and 1.22 respectively).

Ethnicity Classifications

Ethnicity was extracted from electronic health records retrospectively. Ethnicity coding aligned with the UK National Health Census categories and subsequently reallocated into five groups: White, Black, South Asian, East Asian and Other or Not Stated. This process is described in Supplementary Table S1.

Data Collection

Data were extracted retrospectively from electronic health records at each participating site using local clinical informatics pathways. The data extraction process involved sequential identification, linkage and cleaning steps, but was provided as a guide, as sites would have different computer systems and personnel.

First, all individuals with mGFR tests were identified and filtered according to inclusion and exclusion criteria. Secondly, the dataset was linked to serum creatinine results within 30 days of the mGFR result and filtered again as per inclusion / exclusion criteria. Thirdly, additional variables were added (if not done simultaneously in step two) where available. In some sites, this was performed through programmed searches and using statistical software to link the datasets. At other sites this was performed manually.

Finally, records were screened for individuals who had opted out of national data use in accordance with the NHS National Data Opt-Out policy. This was conducted as per local information governance pathways. The final datasets were de-identified and transferred centrally for analysis.

Data extraction by two authors was not performed and likely would be unfeasible given the variability in sample sizes, resource and personnel available between sites.

Data source and validity

mGFR, serum creatinine and serum albumin concentrations were extracted from United Kingdom National Health Service (NHS) electronic health records at participating sites. These were clinically reported laboratory or nuclear medicine department measurements generated as part of routine NHS care and used for clinical decision-making. Demographic and clinical variables, such as ethnicity, sex and referral specialty, were extracted from routinely coded electronic health records.

Data were extracted by the research team, given study constraints which included the multicentre retrospective design with utilisation of multiple electronic health records systems and variable informatic pathways depending on the site. Each participating site extracted data based on local informatic pathways. Data extracted was reviewed by the local study team, to determine if they remained in line with the inclusion and exclusion criteria. Individual values could not be independently remeasured or recoded for research purposes, and residual data limitations may remain because of between-site variation in measurement protocols, coding completeness, local electronic health record systems and extraction pathways.

Measured glomerular filtration rate (mGFR)

mGFR was performed as part of routine clinical care rather than for the purposes of this study. As this was a retrospective analysis, the specific indication for mGFR was not reliably extractable, due to unstructured data formats and variability between sites. We therefore used referral specialty as a surrogate marker for clinical setting and indication.

The two main referral groups were haematology / oncology and nephrology. Typically, haematology / oncology referrals reflect the need for accurate kidney function assessment for chemotherapy dosing (using absolute mGFR; i.e. mL/min) or transplant workup. Nephrology referrals typically include prospective kidney donor assessments or clinical concerns regarding creatinine-based eGFR.

**Supplementary Figures**

**Supplementary Figure 1**. Illustrates the sites involved in the AIM CKD UK with geographical representation.

**Supplementary Figure S2.** Q-Q plots assessing normality of paired differences for each eGFR equation comparison to evaluate the assumption of normality for the repeated-measures ANOVA.

Mauchly's test indicated a violation of sphericity (W=0.002, p<0.001); Greenhouse-Geisser correction was therefore applied (ε=0.322). Following correction, the repeated measures ANOVA remained statistically significant (F(1.29, 20456)=7175.49, p<0.001), confirming a significant difference in eGFR difference between equations. Departure from normality was observed particularly when MDRD-3v was included; a Friedman test with Nemenyi post-hoc analysis was performed as a non-parametric sensitivity analysis and did not alter the direction or statistical significance of findings.

**Supplementary Figure S3.** Illustrates the performance of each eGFR equation stratified by ethnicity for (A) difference using faceted density plots, (B) precision and (C) P30 using bar charts.

(A)

**Mean Difference	(B) Precision	(C) P30**

*(A) Mean Difference is displayed as faceted density plots for each eGFR equation and stratified for ethnicity. Density plots are stratified by ethnicity group. The red dashed line represents the overall mean difference for the subgroup. The blue dashed line represents the overall mean difference for the whole cohort. (B) Standard deviation of the mean difference (precision) plotted against eGFR equation is illustrated as a bar chart with stratification for ethnicity. (C) P30 is plotted against eGFR equation and illustrated as a bar chart with stratification for ethnicity.*

**Supplementary Figure S4.** Lin’s concordance correlation coefficients for eGFR equations stratified by referral specialty.

*Agreement categories are defined by  Landis and Koch as  follows: No agreement < 0, Slight agreement: 0 – 0.2;  Fair agreement: 0.21 – 0.4 ; Moderate agreement: 0.41 – 0.6: Substantial agreement: 0.61 – 0.8; Almost perfect agreement: 0.81 – 1.0.*

**Supplementary Figure S5.** Plots evaluating the assumptions of linearity, normality, homoscedasticity and leverage for each eGFR bias linear regression model.

**Supplementary Figure S6.** Mean Difference, Precision (SD) and Accuracy (P30) stratified by sex for each eGFR equation.

(A) **Mean Difference	(B) Precision	(C) P30**

*(A) Mean Difference is displayed as faceted density plots for each eGFR equation and stratified for sex. Male plots are shaded red and female plots are shaded in turquoise. The red dashed line represents the overall mean difference for the subgroup. The blue dashed line represents the overall mean difference for the whole cohort. (B) Standard deviation of the mean difference (precision) plotted against eGFR equation is illustrated as a bar chart with stratification for sex. (C) P30 is plotted against eGFR equation and illustrated as a bar chart with stratification for sex.*

**Supplementary Figure S7.** Illustrates the trends of (A) Mean difference, (B) Precision and (C) P30 of each eGFR equation when stratified by BMI.

(A) **Mean Difference	(B) Precision	(C) P30**

*(A) Mean difference plotted against BMI as a continuous variable using a LOESS line of best fit. (B) Standard deviation (precision) is plotted as a bar chart against predefined BMI bands for each eGFR equation. (C) P30 (accuracy) is plotted against BMI as a continuous variables using a LOESS line of best fit with 95% confidence intervals shaded in a similar colour, for each eGFR equation.*

**Supplementary Figure S8.** Mean Difference, Precision (SD) and Accuracy (P30) stratified by serum albumin concentration for each eGFR equation.

(A) **Mean Difference	(B) Precision	(C) P30**

*(A) Mean difference plotted against serum albumin concentration as a continuous variable using a LOESS line of best fit. (B) Standard deviation (precision) is plotted as a bar chart against predefined serum albumin concentration bands for each eGFR equation. (C) P30 (accuracy) is plotted against serum albumin concentration as a continuous variables using a LOESS line of best fit with 95% confidence intervals shaded in a similar colour, for each eGFR equation.*

**Supplementary Figure S9.** Mean Difference, Precision (SD) and Accuracy (P30) stratified by serum creatinine concentration for each eGFR equation.

(A)

**Mean Difference	(B) Precision	(C) P30**

*(A) Mean difference plotted against serum creatinine concentration as a continuous variable using a LOESS line of best fit. (B) Standard deviation (precision) is plotted as a bar chart against predefined serum albumin concentration bands for each eGFR equation. (C) P30 (accuracy) is plotted against serum creatinine concentration as a continuous variables using a LOESS line of best fit with 95% confidence intervals shaded in a similar colour, for each eGFR equation.*

**Supplementary Tables**

**Supplementary Table S1.** Reports UK National Census Ethnicity Categories and how these are re-allocated into the ethnicity groups in the AIM CKD UK analysis.

| **AIM CKD UK Ethnicity Groups** | **UK National Census Ethnicity Categories** |
| --- | --- |
| White | English, Welsh, Scottish, Northern Irish or British |
|  | Irish |
|  | Gypsy or Irish Traveller |
|  | Roma |
|  | Any other White background |
| Black | Caribbean |
|  | African |
|  | Any other Black background |
| South Asian | Bangladeshi |
|  | Indian |
|  | Pakistani |
| East Asian | Chinese |
|  | Any other Asian background* |
| Other or Not Stated | White and Asian |
|  | White and Black African |
|  | White and Black Caribbean |
|  | Other mixed or multiple ethnic groups |
|  | Arab |
|  | Any other Asian background** |
|  | Any other ethnic group |
|  | Not stated / Does not apply |

**Specific East Asian ethnic group reported (e.g. Japanese / Vietnamese).*

*** No specific ethnic group reported.*

**Supplementary Table S2.** Summary of the eGFR equation formulae, including year published, origin of development cohorts, ethnicity background and cohort sizes. ^6,7,11,12,18^

| **Abbreviation of GFR Equation** | **Year** | **Cohort Origin** | **Cohort Size** | **GFR Equation Formula** |
| --- | --- | --- | --- | --- |
| MDRD-3v | 1999 | USA  Multiethnic | 1628 | 175 × standardized Cr −1.154 × age−0.203 × 0.742 [if female] |
| CKD-EPI_2009_ | 2009 | USA  Multiethnic | 8254 | 141 x min(Cr /κ, 1)^α^ x max(Cr /κ, 1)^-1.209^ x 0.993^Age^ x 1.018 [if female] |
| CKD-EPI_2021_^i^ | 2021 | USA  Multiethnic | 8254 | 142 x min(Cr /κ, 1)^α^ x max(Cr /κ, 1)^-1.200^ x 0.994^Age^  x 1.012 [if female] |
| LMR^ii^ | 2011 | White Swedish | 850 | eX–0·0158×Age+0·438×ln(Age) |
| EKFC_Cr_ ^iii^ | 2021 | White European | 11251 | Age_________Cr/Q_______Equation 2-40y <1 107.3 x (Cr/Q)^-0.322^  2-40y ≥1 107.3 x (Cr/Q)^-1.132^ >40y ≥1 107.3 x (Cr/Q)^-0.322^ x 0.990^(Age -40)^ >40y ≥1 107.3 x (Cr/Q)^-1.132^ x 0.990^(Age -40)^ |

*The ethnicity coefficient has been removed from the MDRD and CKD-EPI_2009_ equations.*

*^i^ α = -0.241 (females) or -0.302 (males); κ = 0.7mg/dL or 62 µmol/L (females) or 0.9mg/dL or 80µmol/L (males)*

*^ii^ Female Cr<150: X = 2·50+0·0121 × (150– Cr)*

*Female Cr ≥150: X = 2·50–0·926 × ln(Cr /150)*

*Male Cr <180: X = 2·56+0·00968 × (180–Cr)*

*Male Cr ≥180: X = 2·56–0·926 × ln(Cr /180)*

*^iii^ Q values correspond to the median Cr values for the age- and sex-specific populations (Male = 0.9 ; Female = 0.7).*

**Supplementary Table S3**. Describes the medications used to model the potential impact of prescribing eligibility in the AIM CKD UK cohort. Indications for each drug use as per National Institute for Health and Care Excellence (NICE) guidance with upper and lower GFR thresholds and associated rationale for use are included.

| **Drug** | **Indications** | **Lower GFR threshold** | **Upper GFR threshold** | **Rationale for thresholds** |
| --- | --- | --- | --- | --- |
| Dapagliflozin *(SGLT2i)* | Proteinuric kidney disease, Heart failure, Diabetes | 25 | 75 | - Avoid initiation if GFR below 25. - Start if GFR between 25 - 75 |
| Empagliflozin *(SGLT2i)* | Proteinuric kidney disease, Heart failure, Diabetes | 25 | 45 | - Avoid initiation below 25. - Start if GFR between 25-45. |
| Empagliflozin *(SGLT2i)* | Proteinuric kidney disease, Heart failure, Diabetes | 45 | 90 | - Start if GFR between 45-90 and uACR > 22.6 or diabetic. |
| Ramipril *(ACEi)* | Hypertension, Heart disease, CKD, Diabetes | 30 | - | - Reduce dose if GFR < 30 |
| Fineronone *(non-steroidal mineralocorticoid antagonist)* | Proteinuric diabetic kidney disease | 25 | 60 | - Avoid initiation if GFR below 25. - Start if GFR between 25-60. |
| Metformin *(Biguanide)* | Diabetes | 30 | - | - Stop if GFR < 30 |

**Supplementary Table S4**. Demographic and agreement metrics for each eGFR equation stratified by time periods.

| **eGFR Equation** | **Q1 (2009-2011)** | **Q2 (2012 – 2014)** | **Q3 (2015 – 2017)** | **Q4 (2018 – 2020)** | **Q5 (2021-2022)** |
| --- | --- | --- | --- | --- | --- |
| Count, n (%) | 1343 (8.5) | 2891 (18.2) | 3441 (21.7) | 4268 (26.9) | 3936 (24.8) |
| Mean age ± SD, years | 51.1 ± 15.5 | 53 ± 15.4 | 54.4 ± 15.5 | 53.9 ± 16.0 | 55.6 ± 15.6 |
| Male Sex, n (%) | 773 (57.6) | 1717 (59.4) | 2044 (59.4) | 2336 (54.7) | 2023 (51.4) |
| White ethnicity, n (%) | 911 (67.8) | 1933 (66.9) | 2167 (63.0) | 2628 (61.6) | 2246 (57.1) |
| Black ethnicity, n (%) | 43 (3.2) | 143 (4.9) | 201 (5.8) | 213 (5.0) | 240 (6.1) |
| South Asian ethnicity, n (%) | 67 (5.0) | 138 (4.8) | 185 (5.4) | 215 (5.0) | 286 (7.3) |
| East Asian ethnicity, n (%) | 3 (0.2) | 9 (0.3) | 15 (0.4) | 18 (0.4) | 23 (0.6) |
| Other or Not Stated ethnicity, n (%) | 319 (23.8) | 668 (23.1) | 873 (25.4) | 1194 (28.0) | 1141 (29.0) |
| Haematology & Oncology | 835 (62.2) | 1856 (64.2) | 2068 (60.1) | 2679 (62.8) | 2361 (60.0) |
| Nephrology | 379 (28.2) | 589 (20.4) | 737 (21.4) | 796 (18.7) | 615 (15.6) |
| Hepatology | 32 (2.4) | 50 (1.7) | 85 (2.5) | 122 (2.9) | 76 (1.9) |
| Other or Not Stated Referral Specialty | 97 (7.2) | 396 (13.7) | 551 (16.0) | 671 (15.7) | 884 (22.5) |
| CKD stage 3-5 | 384 (28.6) | 606 (21.0) | 859 (25.0) | 963 (22.6) | 836 (21.2) |
| Mean Corrected mGFR ± SD, ml/min/1.73m^2^ | 75.0 ± 26.0 | 78.4 ± 24.3 | 75.2 ± 23.6 | 77.7 ± 23.9 | 77.9 ± 23.2 |
| **Mean Difference ± Standard Deviation, mL/min/1.73m^2^** | | | | | |
| MDRD | 5.0 ± 20.2 | 11.9 ± 26.2 | 10.2 ± 22.5 | 8.9 ± 22.1 | 7.8 ± 22.8 |
| CKD-EPI 2009 | 8.3 ± 15.5 | 11.7 ± 16.7 | 10.9 ± 15.8 | 9.9 ± 15.3 | 8.6 ± 16.0 |
| CKD-EPI 2021 | 11.5 ± 15.4 | 14.8 ± 16.5 | 14.2 ± 15.8 | 13.2 ± 15.2 | 12.0 ± 15.9 |
| EKFC | 4.3 ± 14.3 | 6.7 ± 15.6 | 6.1 ± 14.7 | 4.9 ± 14.1 | 3.6 ± 15.0 |
| LM Revised | -0.1 ± 14.5 | 2.8 ± 16.2 | 2.4 ± 15.0 | 1.1 ± 14.5 | 0.1 ± 15.2 |
| **Median Difference (Interquartile Range), mL/min/1.73m^2^** | | | | | |
| MDRD | 2.8 (-6.7 to 14.2) | 8.2 (-3.3 to 23.3) | 6.9 (-3.9 to 21.0) | 6.3 (-4.8 to 19.2) | 5.4 (-6.5 to 18.4) |
| CKD-EPI 2009 | 7.5 (-1.8 to 17.9) | 11.4 (0.9 to 22.1) | 10.3 (0.5 to 20.6) | 9.7 (0.0 to 19.6) | 8.8 (-1.4 to 18.7) |
| CKD-EPI 2021 | 10.9 (1.4 to 21.4) | 14.7 (3.9 to 25.1) | 13.8 (3.9 to 24.1) | 13.1 (3.4 to 23.0) | 12.3 (2.0 to 22.2) |
| EKFC | 3.9 (-4.5 to 12.9) | 6.3 (-2.9 to 16.4) | 5.6 (-3.3 to 15.0) | 4.8 (-4.1 to 13.8) | 3.7 (-5.6 to 12.9) |
| LM Revised | 0.0 (-8.7 to 8.4) | 2.3 (-7.1 to 13.0) | 1.8 (-7.0 to 11.6) | 1.3 (-8.1 to 10.1) | 0.1 (-9.2 to 9.2) |
| **P30 (95% Confidence Intervals), %** | | | | | |
| MDRD | 79.4 [77.2 to 81.5] | 69.2 [67.5 to 70.9] | 71.7 [70.2 to 73.2] | 74.8 [73.5 to 76.1] | 75.0 [73.7 to 76.4] |
| CKD-EPI 2009 | 78.8 [76.6 to 81.0] | 73.0 [71.4 to 74.6] | 74.2 [72.8 to 75.7] | 76.8 [75.5 to 78.1] | 78.0 [76.7 to 79.3] |
| CKD-EPI 2021 | 73.5 [71.1 to 75.9] | 67.5 [65.7 to 69.2] | 68.0 [66.4 to 69.5] | 70.3 [68.9 to 71.7] | 72.7 [71.3 to 74.1] |
| EKFC | 84.7 [82.8 to 86.7] | 80.7 [79.3 to 82.1] | 82.4 [81.1 to 83.7] | 85.2 [84.1 to 86.2] | 84.7 [83.6 to 85.8] |
| LM Revised | 86.3 [84.5 to 88.1] | 83.4 [82.0 to 84.8] | 85.8 [84.6 to 87.0] | 87.3 [86.3 to 88.3] | 86.8 [85.8 to 87.9] |

# *Equation abbreviations: MDRD = Modified Diet in Renal Disease; CKD-EPI = Chronic Kidney Disease Epidemiology Collaboration; EKFC = European Kidney Function Consortium; LM = Lund-Malmo.*

**Supplementary Table S5.** Mean difference ± standard deviation and P30 (%) for biological sex, mGFR CKD stages, mGFR filtration markers and time between mGFR and eGFR tests.

|  | MDRD-3v | CKD-EPI_2009_ | CKD-EPI_2021_ | EKFC | LMR |
| --- | --- | --- | --- | --- | --- |
| Mean Difference ± Standard Deviation (mL/min/1.73m^2^) | | | | | |
| Male | 8.9 ± 23.3 | 9.1 ± 16.1 | 12.5 ± 15.9 | 4.4 ± 14.9 | -0.6 ± 15.1 |
| Female | 9.4 ± 22.8 | 11.1 ±15.6 | 14.3 ± 15.6 | 6.0 ± 14.6 | 3.9 ± 14.8 |
| mGFR (mL/min/1.73m^2^):  ≥ 90 60 – 89 30 – 59 < 29 | 4.2 ± 27.1  11.4 ± 21.7  12.1 ± 18.2  8.4 ± 14.1 | 3.5 ± 15.3 13.0 ± 14.8 13.6 ± 16.3 9.0 ± 14.8 | 6.1 ± 14.4 16.8 ± 14.5 17.2 ± 16.8 10.9 ± 15.6 | -2.4 ± 13.6 8.0 ± 13.6 10.2 ± 14.7 8.5 ± 13.5 | -7.8 ± 14.1 4.4 ± 13.2 8.5 ± 14.2 6.7 ± 13.0 |
| mGFR Filtration Marker:  ^99m^Technetium-DTPA ^51^Chromium-EDTA Not Stated | 8.5 ± 22.9  10.2 ± 23.5 2.8 ± 18.4 | 9.0 ± 15.8 10.9 ± 16.0 8.1 ± 14.9 | 12.4 ± 15.7 14.1 ± 15.9 11.5 ± 14.7 | 4.0 ± 14.7 6.1 ± 14.9 3.6 ± 13.8 | 0.5 ±15.0 2.3 ± 15.2 -1.7 ± 13.9 |
| Time between Tests:  Same Day  1-7 Days 8-30 Days | 8.3 ± 23.1 12.7 ± 23.7 7.6 ± 22.0 | 9.5 ± 15.9 11.7 ± 15.5 9.3 ± 16.3 | 12.8 ± 15.7 15.0 ± 15.4 12.7 ± 16.4 | 4.7 ± 14.8 6.6 ± 14.4 4.7 ± 15.1 | 0.7 ± 15.1 3.4 ± 14.8 0.8 ± 15.2 |
| P30 (%) | | | | | |
| Male | 73.8 | 77.7 | 72.2 | 84.7 | 87.7 |
| Female | 73.2 | 73.8 | 67.5 | 82.2 | 83.9 |
| mGFR (mL/min/1.73m^2^):  ≥ 90 60 – 89 30 – 59 < 29 | 83.0 74.0 60.7 52.1 | 94.5 73.8 55.2 50.5 | 94.5 64.9 46.5 45.9 | 97.5 84.8 63.7 51.4 | 95.9 89.5 66.5 60.2 |
| mGFR Filtration Marker:  ^99m^Technetium-DTPA ^51^Chromium-EDTA Not Stated | 74.2 71.6 87.2 | 77.2 74.0 86.4 | 71.6 67.9 80.6 | 84.7 81.9 91.5 | 86.7 84.8 93.0 |
| Time between Tests:  Same Day  1-7 Days 8-30 Days | 75.5 68.8 73.5 | 77.4 73.2 75.2 | 71.9 66.3 69.6 | 84.5 81.8 83.0 | 86.9 84.4 85.4 |

# **Supplementary Table S6.** Mean difference, median difference and P30 for each eGFR equation stratified by site.

| **eGFR Equation** | **BRAD** | **GGC** | **KCH** | **LTH** | **MRI** | **NCA** | **OUH** | **RLH** | **SGH** | **UHB** | **UHL** |
| --- | --- | --- | --- | --- | --- | --- | --- | --- | --- | --- | --- |
| Mean Difference ± Standard Deviation, mL/min/1.73m^2^ | | | | | | | | | | | |
| MDRD | -3.4 ± 17.8 | 7.2 ± 19.4 | 11.4 ± 23.5 | 9.7 ± 25.8 | -4.6 ± 16.0 | 8.0 ± 17.4 | 11.7 ± 25.7 | 1.4 ± 21.8 | 18.2 ± 24.4 | 7.1 ± 22.0 | 3.0 ± 18.3 |
| CKD-EPI 2009 | 2.0 ± 16.5 | 10.6 ± 15.4 | 11.2 ± 16.3 | 10.5 ± 16.3 | 1.9 ± 14.9 | 13.4 ± 16.5 | 10.5 ± 15.6 | 3.6 ± 16.8 | 14.4 ± 15.2 | 8.5 ± 15.1 | 8.3 ± 14.7 |
| CKD-EPI 2021 | 5.4 ± 16.5 | 13.7 ± 15.2 | 14.5 ± 16.2 | 13.3 ± 15.6 | 5.1 ± 14.9 | 16.8 ± 16.5 | 13.5 ± 15.0 | 7.4 ± 16.9 | 17.8 ± 15.2 | 11.8 ± 15.0 | 11.7 ± 14.5 |
| EKFC | -2.3 ± 15.4 | 6.0 ± 14.4 | 6.5 ± 15.3 | 4.5 ± 14.4 | -0.8 ± 13.7 | 9.3 ± 16.0 | 4.6 ± 14.4 | -0.9 ± 15.9 | 8.6 ± 14.3 | 3.9 ± 14.0 | 3.8 ± 13.6 |
| LM Revised | -6.8 ± 14.4 | 0.8 ± 14.4 | 3.2 ± 15.5 | 0.2 ± 14.9 | -5.5 ± 13.5 | 4.3 ± 15.4 | 1.1 ± 14.8 | -4.1 ± 16.1 | 6.5 ± 14.5 | -0.3 ± 14.5 | -1.5 ± 13.8 |
| Median Difference (Interquartile Range), mL/min/1.73m^2^ | | | | | | | | | | | |
| MDRD | -2.7 (-15.9 to 7.7) | 5.3 (-4.6 to 16.0) | 9.0 (-3.0 to 22.4) | 5.1 (-5.6 to 19.2) | -4.2 (-14.0 to 4.4) | 5.9 (-1.4 to 14.9) | 6.9 (-3.9 to 22.7) | -0.4 (-12.6 to 12.0) | 14.9 (3.0 to 29.4) | 4.5 (-5.9 to 16.8) | 1.6 (-8.0 to 12.2) |
| CKD-EPI 2009 | 3.5 (-7.5 to 11.8) | 10.1 (0.7 to 20.0) | 10.9 (0.7 to 21.9) | 9.9 (0.1 to 20.0) | 2.2 (-6.8 to 12.1) | 12.2 (2.9 to 20.6) | 10.6 (0.8 to 20.2) | 2.9 (-7.5 to 14.9) | 13.9 (4.9 to 23.8) | 7.8 (-1.4 to 17.9) | 7.7 (-1.0 to 17.7) |
| CKD-EPI 2021 | 6.2 (-4.3 to 15.4) | 13.3 (3.8 to 23.2) | 14.5 (4.3 to 25.1) | 12.9 (3.4 to 22.5) | 5.6 (-3.6 to 15.4) | 15.9 (6.4 to 24.1) | 13.9 (3.9 to 23.3) | 6.8 (-3.5 to 18.7) | 17.7 (8.3 to 27.5) | 11.5 (1.9 to 21.2) | 11.1 (2.6 to 20.9) |
| EKFC | -0.5 (-12.2 to 6.8) | 5.7 (-3.0 to 14.5) | 6.0 (-3.5 to 16.4) | 4.2 (-4.3 to 13.9) | -0.0 (-8.9 to 8.2) | 7.8 (0.7 to 15.6) | 4.6 (-4.3 to 13.5) | -1.4 (-11.4 to 9.1) | 8.1 (-0.7 to 17.6) | 3.6 (-4.8 to 12.4) | 3.7 (-4.5 to 12.0) |
| LM Revised | -4.8 (-18.0 to 2.0) | 0.6 (-8.2 to 9.3) | 3.0 (-6.7 to 12.9) | -0.2 (-9.1 to 9.1) | -4.5 (-13.4 to 3.2) | 3.5 (-4.4 to 10.2) | 0.5 (-8.2 to 10.3) | -4.5 (-15.0 to 6.2) | 6.0 (-2.9 to 15.5) | -0.2 (-9.4 to 8.7) | -1.6 (-10.1 to 6.5) |
| P30 (95% Confidence Intervals), % | | | | | | | | | | | |
| MDRD | 85.5 (78.0 to 93.1) | 80.0 (78.2 to 81.9) | 69.9 (68.1 to 71.8) | 77.8 (75.2 to 80.5) | 87.1 (85.0 to 89.2) | 82.7 (78.8 to 86.6) | 73.9 (70.0 to 77.8) | 77.6 (74.8 to 80.4) | 58.3 (56.6 to 60.0) | 76.8 (75.4 to 78.1) | 87.4 (85.1 to 89.6) |
| CKD-EPI 2009 | 89.2 (82.5 to 95.8) | 78.4 (76.6 to 80.3) | 72.4 (70.6 to 74.2) | 79.3 (76.6 to 81.9) | 88.8 (86.8 to 90.7) | 75.4 (71.0 to 79.9) | 79.4 (75.8 to 83.0) | 80.8 (78.2 to 83.4) | 65.2 (63.6 to 66.8) | 78.7 (77.4 to 80.0) | 86.4 (84.0 to 88.7) |
| CKD-EPI 2021 | 84.3 (76.5 to 92.2) | 72.7 (70.7 to 74.7) | 66.1 (64.2 to 68.0) | 76.0 (73.2 to 78.8) | 85.4 (83.3 to 87.6) | 67.6 (62.8 to 72.4) | 74.9 (71.1 to 78.7) | 76.9 (74.2 to 79.7) | 56.9 (55.2 to 58.6) | 73.5 (72.1 to 75.0) | 80.4 (77.7 to 83.1) |
| EKFC | 91.6 (85.6 to 97.5) | 85.5 (83.9 to 87.1) | 80.0 (78.4 to 81.6) | 88.0 (85.8 to 90.0) | 90.8 (89.1 to 92.6) | 85.2 (81.5 to 88.9) | 88.0 (85.1 to 90.8) | 84.6 (82.2 to 87.0) | 76.5 (75.1 to 77.9) | 85.5 (84.4 to 86.7) | 91.6 (89.7 to 93.5) |
| LM Revised | 91.6 (85.6 to 97.5) | 87.9 (86.4 to 89.4) | 83.9 (82.4 to 85.3) | 89.9 (88.0 to 91.8) | 90.9 (89.2 to 92.7) | 89.1 (85.9 to 92.3) | 90.0 (87.3 to 92.7) | 85.9 (83.6 to 88.2) | 80.0 (78.6 to 81.3) | 87.4 (86.3 to 88.5) | 93.0 (91.3 to 94.8) |

# Site abbreviations: BRAD = St Luke’s Hospital, Bradford; GGC = NHS Greater Glasgow and Clyde; KCH = King’s College Hospital NHS Foundation Trust; LTH = Leeds Teaching Hospitals NHS Trust; MRI = Manchester Royal Infirmary, Manchester University Hospitals NHS Foundation Trust; NCA = Royal Salford Hospital, Northern Care Alliance; OUH = Oxford University Hospitals; RLH = Royal London Hospital, Barts Health NHS Trust; SGH = St George’s University Hospital NHS Foundation Trust; UHB = University Hospitals Birmingham NHS Foundation Trust; UHL = University Hospitals Leicester NHS Foundation Trust Equation abbreviations: MDRD = Modified Diet in Renal Disease; CKD-EPI = Chronic Kidney Disease Epidemiology Collaboration; EKFC = European Kidney Function Consortium; LM = Lund-Malmo.

**Supplementary Table S7.** Agreement between eGFR and mGFR for each equation, including using the Black ethnicity coefficient for the MDRD and CKD-EPI 2009 equation in the Black ethnicity subgroup.

| eGFR Equation | Mean difference, mL/min/1.73m^2^ [95% CI] | SD | Median difference, mL/min/1.73m^2^ [95% CI] | IQR, mL/min/1.73m^2^ | RMSE, mL/min/1.73m^2^ | P30, % [95% CI] | 95% LoA  (lower to upper) |
| --- | --- | --- | --- | --- | --- | --- | --- |
| MDRD-3v | 2.8 [1.3 to 4.3] | 22.2 | 0.2 [-1.5 to 1.7] | -11.1 to 15.3 | 22.3 | 74.1 [71.1 to 77.0] | -40.7 to 46.3 |
| MDRD-4v* | 19.2 [17.4 to 21.0] | 26.6 | 14.9 [12.9 to 17.3] | 1.6 to 34.1 | 32.8 | 56.3 [53.0 to 60.0] | -32.9 to 71.4 |
| CKD-EPI_2009_ (AS) | 4.5 [3.3 to 5.7] | 17.9 | 3.3 [2.0 to 5.2] | -6.6 to 16.1 | 18.5 | 77.3 [74.4 to 80.1] | -30.6 to 39.7 |
| CKD-EPI_2009_ (ASE)* | 17.1 [15.8 to 18.5] | 20.3 | 16.8 [15.0 to 18.0] | 3.4 to 30.7 | 26.6 | 60.0 [56.6 to 63.2] | -22.7 to 56.9 |
| CKD-EPI_2021_ | 7.9 [6.7 to 9.1] | 18.0 | 6.9 [5.2 to 8.3] | -3.4 to 19.8 | 19.6 | 74.1 [71.1 to 77.0] | -27.3 to 43.1 |
| EKFC | 0.6 [-0.5 to 1.7] | 16.4 | -0.5 [-1.4 to 0.8] | -9.4 to 10.1 | 16.4 | 82.4 [79.8 to 85.0] | -31.6 to 32.8 |
| LM Revised | -2.6 [-3.7 to -1.5] | 16.3 | -3.5 [-4.4 to -2.3] | -12.8 to 6.8 | 16.5 | 82.7 [80.2 to 85.3] | -34.5 to 29.2 |

# *Equation abbreviations: MDRD = Modified Diet in Renal Disease; CKD-EPI = Chronic Kidney Disease Epidemiology Collaboration; EKFC = European Kidney Function Consortium; LM = Lund-Malmo.*

**Equation uses an ethnicity co-efficient. MDRD = 1.21 ; CKD-EPI 2009 (ASE) = 1.16.*

**Supplementary Table S8**. Demographic and clinical characteristics stratified by referral specialty.

|  | **Haematology & Oncology, N (%)** | **Nephrology, N (%)** | **Hepatology, N (%)** | **Other / Not Stated, N (%)** | **p-value** |
| --- | --- | --- | --- | --- | --- |
| Mean Age ± SD,  years | 55.4 ± 16.0 | 47.2 ± 13.7 | 60.4 ± 14.2 | 56.2 ± 15.6 | < 0.001 |
| Male Sex | 5779 (59.0) | 1564 (50.2) | 233 (63.9) | 1317 (50.7) | < 0.001 |
| Mean corrected mGFR ± SD, mL/min/1.73m^2^ | 77.0 ± 23.8 | 80.4 ± 23.8 | 60.8 ± 25.7 | 75.8 ± 23.5 | < 0.001 |
| Median BMI (IQR), kg/m^2^ | 26.1 (23.0 to 29.8) | 26.7 (23.9 to 30.2) | 24.4 (21.5 to 28.4) | 25.5 (22.4 to 29.4) | < 0.001 |
| Median Serum albumin, g/L | 40 (35 to 44) | 42 (39 to 45) | 37 (32 to 42) | 41 (35 to 45) | < 0.001 |
| **BMI Bands, kg/m^2^** | | | | | |
| <18.5 | 287 (3.7) | 44 (1.65) | 24 (6.7) | 90 (5.4) | < 0.001 |
| 18.5 - 24.9 | 2962 (38.19) | 876 (32.94) | 171 (47.77) | 667 (40.04) |  |
| 25 - 29.9 | 2627 (33.87) | 1042 (39.19) | 110 (30.73) | 534 (32.05) |  |
| 30 - 34.9 | 1212 (15.63) | 523 (19.67) | 43 (12.01) | 258 (15.49) |  |
| 35 - 39.9 | 435 (5.61) | 128 (4.81) | 7 (1.96) | 79 (4.74) |  |
| ≥ 40 | 233 (3) | 46 (1.73) | 3 (0.84) | 38 (2.28) |  |
| **Albumin Bands, g/L** | | | | | |
| 0-20 | 61 (0.62) | 5 (0.16) | 6 (1.66) | 35 (1.42) | < 0.001 |
| 21-25 | 217 (2.22) | 14 (0.46) | 23 (6.35) | 74 (3.01) |  |
| 26-30 | 565 (5.77) | 21 (0.68) | 42 (11.6) | 187 (7.61) |  |
| 31-35 | 1344 (13.74) | 93 (3.03) | 59 (16.3) | 284 (11.55) |  |
| 36-40 | 2657 (27.15) | 766 (24.93) | 87 (24.03) | 443 (18.02) |  |
| 41-45 | 3025 (30.91) | 1335 (43.44) | 86 (23.76) | 728 (29.62) |  |
| 46-50 | 1620 (16.56) | 733 (23.85) | 54 (14.92) | 644 (26.2) |  |
| >50 | 296 (3.03) | 106 (3.45) | 5 (1.38) | 63 (2.56) |  |

# *Equation abbreviations: MDRD = Modified Diet in Renal Disease; CKD-EPI = Chronic Kidney Disease Epidemiology Collaboration; EKFC = European Kidney Function Consortium; LM = Lund-Malmo.*

**Supplementary Table S9***.* Proportion of correctly classified CKD stages across the whole cohort, with stratification for ethnicity and referral specialty.

|  |  | **MDRD-3v** | **CKD-EPI_2009_** | **CKD-EPI_2021_** | **EKFC** | **LMR** |
| --- | --- | --- | --- | --- | --- | --- |
|  | Whole Cohort  (n = 15879) | 60.1  (59.3 to 60.9) | 59.6  (58.9 to 60.4) | 55.4  (54.6 to 56.1) | 64.9  (64.1 to 65.6) | 65.0  (64.2 to 65.7) |
| **Ethnicity:**  Correctly Classified,% (95% CI) | White (n = 9885) | 61.0 (60.1 to 62.0) | 59.7 (58.8 to 60.7) | 55.0 (54.0 to 56.0) | 65.5  64.5 to 66.4) | 66.1 (65.1 to 67.0) |
|  | Black (n = 840) | 55.5 (52.1 to 58.8) | 58.1 (54.7 to 61.4) | 55.4 (52.0 to 58.7) | 61.3 (58.0 to 64.5) | 59.5 (56.2 to 62.8) |
|  | South Asian (n = 891) | 57.9 (54.6 to 61.1) | 54.9 (51.6 to 58.1) | 50.7 (47.5 to 54.0) | 60.6 (57.4 to 63.8) | 63.1 (60.0 to 66.2) |
|  | Other/Not Stated (n = 4195) | 59.2 (57.7 to 60.7) | 60.6 (59.1 to 62.0) | 57.1 (55.6 to 58.6) | 65.1 (63.6 to 66.5) | 63.8 (62.4 to 65.3) |
| **Referral Team:**  Correctly Classified,% (95% CI) | Nephrology (n = 3116) | 64.1 62.4 to 65.8) | 64.5 (62.8 to 66.2) | 62.7 (61.0 to 64.4) | 67.6 (65.9 to 69.2) | 66.3 64.7 (68.0) |
|  | Haematology / Oncology (n = 9799) | 59.8 (58.8 to 60.8) | 58.7 (57.7 to 59.7) | 53.8 (52.8 to 54.8) | 64.2 (63.2 to 65.1) | 64.9 (63.9 to 65.8) |
|  | Hepatology (n = 365) | 57.5 (52.4 to 62.5) | 56.7 (51.6 to 61.7) | 54.0 (48.8 to 59.0) | 63.8 (58.8 to 68.6) | 62.5 (57.4 to 67.3) |

**Supplementary Table S10.** Number of participants who would fall within the treatment ranges (for initiation, cessation and dose adjustment) based on their mGFR result. Results are stratified by eGFR equation and ethnicity. Each table is divided into those within range, above the GFR threshold and below the GFR threshold. Tables included are for (A) Dapagliflozin (25 - 75 mL/min/1.73m^2^), (B) Empagliflozin (25 - 45 mL/min/1.73m^2^), (C) Empagliflozin (45 – 90 mL/min/1.73m^2^), (D) Fineronone (25 – 60 mL/min/1.73m^2^) and (E) Ramipril and Metformin (≥ 30 mL/min/1.73m^2^).

# (A) Dapagliflozin (25 – 75 mL/min/1.73m^2^)

| **eGFR Equation** | **Count** | **Mean Age ± SD** | **Median BMI (IQR)** | **White, N (%)** | **Black, N (%)** | **South Asian, N (%)** | **East Asian, N (%)** | **Other/Not Stated, N (%)** |
| --- | --- | --- | --- | --- | --- | --- | --- | --- |
| **Within Range** | | | | | | | | |
| mGFR | 6586 | 62.5 ± 13.0 | 26.4 (23.2–30.3) | 4283 | 380 | 344 (100.0%) | 21 (100.0%) | 1558 (100.0%) |
| **Above GFR Threshold** | | | | | | | | |
| MDRD-3v | 2469 | 61.9 ± 12.9 | 25.4 (22.5–29.8) | 1579 (36.9%) | 103 (27.1%) | 149 (43.3%) | 5 (23.8%) | 633 (40.6%) |
| CKD-EPI_2009_ | 2980 | 60.6 ± 12.9 | 25.7 (22.7–30.0) | 1912 (44.6%) | 128 (33.7%) | 181 (52.6%) | 6 (28.6%) | 753 (48.3%) |
| CKD-EPI_2021_ | 3433 | 61.3 ± 12.9 | 25.7 (22.7–30.0) | 2192 (51.2%) | 152 (40.0%) | 202 (58.7%) | 8 (38.1%) | 879 (56.4%) |
| EKFC | 2393 | 58.2 ± 12.4 | 25.7 (22.6–30.2) | 1534 (35.8%) | 97 (25.5%) | 151 (43.9%) | 2 (9.5%) | 609 (39.1%) |
| LMR | 1933 | 57.3 ± 12.4 | 25.8 (22.5–30.2) | 1245 (29.1%) | 79 (20.8%) | 122 (35.5%) | 0 (0.0%) | 487 (31.3%) |
| **Below GFR Threshold** | | | | | | | | |
| MDRD-3v | 69 | 59.2 ± 13.6 | 27.1 (24.6–32.1) | 52 (1.2%) | 6 (1.6%) | 2 (0.6%) | 0 (0.0%) | 9 (0.6%) |
| CKD-EPI_2009_ | 60 | 62.8 ± 13.2 | 27.1 (24.6–30.9) | 46 (1.1%) | 4 (1.1%) | 2 (0.6%) | 0 (0.0%) | 8 (0.5%) |
| CKD-EPI_2021_ | 48 | 61.8 ± 13.1 | 27.1 (25.1–30.7) | 37 (0.9%) | 3 (0.8%) | 2 (0.6%) | 0 (0.0%) | 6 (0.4%) |
| EKFC | 60 | 65.6 ± 13.2 | 27.2 (25.2–31.6) | 47 (1.1%) | 4 (1.1%) | 1 (0.3%) | 0 (0.0%) | 8 (0.5%) |
| LMR | 111 | 65.3 ± 13.9 | 27.5 (24.8–32.3) | 82 (1.9%) | 9 (2.4%) | 3 (0.9%) | 0 (0.0%) | 17 (1.1%) |

(B) Empagliflozin (25 – 45 mL/min/1.73m^2^)

| **eGFR Equation** | **Count** | **Mean Age ± SD** | **Median BMI (IQR)** | **White, N (%)** | **Black, N (%)** | **South Asian, N (%)** | **East Asian, N (%)** | **Other/ NS, N (%)** |
| --- | --- | --- | --- | --- | --- | --- | --- | --- |
| **Within Range** | | | | | | | | |
| mGFR | 1438 | 64.6 ± 13.3 | 26.9 (23.5–31.1) | 955 | 77 | 84 | 7 | 315 |
| **Above GFR Threshold** | | | | | | | | |
| MDRD-3v | 696 | 66.6 ± 12.4 | 26.7 (23.2–30.9) | 446 (46.7%) | 29 (37.7%) | 47 (56.0%) | 4 (57.1%) | 170 (54.0%) |
| CKD-EPI_2009_ | 744 | 65.2 ± 13.0 | 26.8 (23.2–30.9) | 480 (50.3%) | 30 (39.0%) | 50 (59.5%) | 4 (57.1%) | 180 (57.1%) |
| CKD-EPI_2021_ | 858 | 65.3 ± 13.0 | 26.8 (23.1–30.9) | 557 (58.3%) | 35 (45.5%) | 58 (69.0%) | 5 (71.4%) | 203 (64.4%) |
| EKFC | 683 | 64.1 ± 12.8 | 26.8 (23.2–30.9) | 441 (46.2%) | 26 (33.8%) | 48 (57.1%) | 2 (28.6%) | 166 (52.7%) |
| LMR | 646 | 65.2 ± 12.5 | 26.8 (23.2–30.9) | 409 (42.8%) | 26 (33.8%) | 47 (56.0%) | 2 (28.6%) | 162 (51.4%) |
| **Below GFR Threshold** | | | | | | | | |
| MDRD-3v | 63 | 57.7 ± 13.1 | 27.1 (24.5–32.3) | 47 (4.9%) | 6 (7.8%) | 2 (2.4%) | 0 (0.0%) | 8 (2.5%) |
| CKD-EPI_2009_ | 54 | 61.5 ± 13.0 | 27.1 (24.5–31.8) | 41 (4.3%) | 4 (5.2%) | 2 (2.4%) | 0 (0.0%) | 7 (2.2%) |
| CKD-EPI_2021_ | 42 | 60.0 ± 12.6 | 27.3 (24.9–31.4) | 32 (3.4%) | 3 (3.9%) | 2 (2.4%) | 0 (0.0%) | 5 (1.6%) |
| EKFC | 54 | 64.6 ± 13.3 | 27.5 (25.0–32.1) | 42 (4.4%) | 4 (5.2%) | 1 (1.2%) | 0 (0.0%) | 7 (2.2%) |
| LMR | 103 | 64.6 ± 14.0 | 27.8 (24.9–33.0) | 76 (8.0%) | 9 (11.7%) | 3 (3.6%) | 0 (0.0%) | 15 (4.8%) |

(C) Empagliflozin (45 – 90 mL/min/1.73m^2^)

| **eGFR Equation** | **Count** | **Mean Age ± SD** | **Median BMI (IQR)** | **White, N (%)** | **Black, N (%)** | **South Asian, N (%)** | **East Asian, N (%)** | **Other/ NS, N (%)** |
| --- | --- | --- | --- | --- | --- | --- | --- | --- |
| **Within Range** | | | | | | | | |
| mGFR | 9326 | 57.9 ± 13.9 | 26.2 (23.1–29.9) | 5903 | 546 | 501 | 35 | 2341 |
| **Above GFR Threshold** | | | | | | | | |
| MDRD-3v | 3048 | 55.6 ± 14.8 | 25.1 (22.0–28.7) | 1891 (32.0%) | 129 (23.6%) | 210 (41.9%) | 11 (31.4%) | 807 (34.5%) |
| CKD-EPI_2009_ | 3960 | 52.6 ± 13.4 | 25.4 (22.4–29.4) | 2474 (41.9%) | 168 (30.8%) | 260 (51.9%) | 12 (34.3%) | 1046 (44.7%) |
| CKD-EPI_2021_ | 4865 | 54.6 ± 13.8 | 25.5 (22.6–29.4) | 3078 (52.1%) | 205 (37.5%) | 304 (60.7%) | 17 (48.6%) | 1261 (53.9%) |
| EKFC | 2688 | 47.5 ± 11.7 | 25.6 (22.5–29.6) | 1615 (27.4%) | 116 (21.2%) | 206 (41.1%) | 9 (25.7%) | 742 (31.7%) |
| LMR | 1815 | 48.6 ± 13.3 | 25.1 (21.9–28.8) | 1072 (18.2%) | 79 (14.5%) | 154 (30.7%) | 6 (17.1%) | 504 (21.5%) |
| **Below GFR Threshold** | | | | | | | | |
| MDRD-3v | 239 | 60.3 ± 14.0 | 27.0 (24.5–30.5) | 130 (2.2%) | 39 (7.1%) | 6 (1.2%) | 2 (5.7%) | 62 (2.6%) |
| CKD-EPI_2009_ | 182 | 64.5 ± 12.9 | 27.2 (24.5–30.6) | 99 (1.7%) | 31 (5.7%) | 4 (0.8%) | 1 (2.9%) | 47 (2.0%) |
| CKD-EPI_2021_ | 120 | 64.8 ± 13.0 | 26.7 (24.6–30.4) | 69 (1.2%) | 21 (3.8%) | 4 (0.8%) | 0 (0.0%) | 26 (1.1%) |
| EKFC | 218 | 67.7 ± 11.8 | 26.7 (24.0–29.8) | 123 (2.1%) | 34 (6.2%) | 4 (0.8%) | 1 (2.9%) | 56 (2.4%) |
| LMR | 264 | 63.3 ± 14.2 | 27.2 (24.5–30.5) | 148 (2.5%) | 44 (8.1%) | 5 (1.0%) | 1 (2.9%) | 66 (2.8%) |

(D) Fineronone (25 – 60 mL/min/1.73m^2^)

| **eGFR Equation** | **Count** | **Mean Age ± SD** | **Median BMI (IQR)** | **White, N (%)** | **Black, N (%)** | **South Asian, N (%)** | **East Asian, N (%)** | **Other/ NS, N (%)** |
| --- | --- | --- | --- | --- | --- | --- | --- | --- |
| **Within Range** | | | | | | | | |
| mGFR | 3476 | 64.7 ± 12.7 | 26.6 (23.2–30.4) | 2277 (100.0%) | 195 (100.0%) | 192 (100.0%) | 15 (100.0%) | 797 (100.0%) |
| **Above GFR Threshold** | | | | | | | | |
| MDRD-3v | 1484 | 65.9 ± 12.1 | 25.8 (22.5–29.9) | 948 (41.6%) | 64 (32.8%) | 94 (49.0%) | 7 (46.7%) | 371 (46.5%) |
| CKD-EPI_2009_ | 1656 | 64.6 ± 12.5 | 26.0 (22.6–30.0) | 1065 (46.8%) | 68 (34.9%) | 107 (55.7%) | 8 (53.3%) | 408 (51.2%) |
| CKD-EPI_2021_ | 1892 | 65.0 ± 12.4 | 26.1 (22.7–30.0) | 1222 (53.7%) | 81 (41.5%) | 119 (62.0%) | 8 (53.3%) | 462 (58.0%) |
| EKFC | 1405 | 63.2 ± 12.3 | 25.9 (22.5–29.9) | 902 (39.6%) | 53 (27.2%) | 94 (49.0%) | 7 (46.7%) | 349 (43.8%) |
| LMR | 1361 | 63.4 ± 12.0 | 26.0 (22.6–30.0) | 875 (38.4%) | 54 (27.7%) | 95 (49.5%) | 5 (33.3%) | 332 (41.7%) |
| **Below GFR Threshold** | | | | | | | | |
| MDRD-3v | 66 | 58.8 ± 13.8 | 27.1 (24.5–32.2) | 49 (2.2%) | 6 (3.1%) | 2 (1.0%) | 0 (0.0%) | 9 (1.1%) |
| CKD-EPI_2009_ | 57 | 62.6 ± 13.5 | 27.1 (24.5–31.2) | 43 (1.9%) | 4 (2.1%) | 2 (1.0%) | 0 (0.0%) | 8 (1.0%) |
| CKD-EPI_2021_ | 45 | 61.4 ± 13.4 | 27.1 (24.8–31.0) | 34 (1.5%) | 3 (1.5%) | 2 (1.0%) | 0 (0.0%) | 6 (0.8%) |
| EKFC | 57 | 65.5 ± 13.5 | 27.2 (24.9–32.0) | 44 (1.9%) | 4 (2.1%) | 1 (0.5%) | 0 (0.0%) | 8 (1.0%) |
| LMR | 108 | 65.3 ± 14.1 | 27.6 (24.6–32.5) | 79 (3.5%) | 9 (4.6%) | 3 (1.6%) | 0 (0.0%) | 17 (2.1%) |

(E) Ramipril / Metformin (≥ 30 mL/min/1.73m^2^)

| eGFR Equation | Count | Mean Age ± SD | Median BMI (IQR) | White, N (%) | Black, N (%) | South Asian, N (%) | East Asian, N (%) | Other/ NS, N (%) |
| --- | --- | --- | --- | --- | --- | --- | --- | --- |
| **Within Range** | | | | | | | | |
| mGFR | 15336 | 53.8 ± 15.7 | 26.1 (23.0–29.8) | 9537 | 810 | 830 | 66 | 4093 |
| **Below GFR Threshold** | | | | | | | | |
| MDRD-3v | 98 | 60.2 ± 14.6 | 27.4 (24.7–32.3) | 65 (0.7%) | 12 (1.5%) | 1 (0.1%) | 1 (1.5%) | 19 (0.5%) |
| CKD-EPI_2009_ | 94 | 62.1 ± 14.9 | 27.2 (24.6–31.8) | 60 (0.6%) | 13 (1.6%) | 1 (0.1%) | 1 (1.5%) | 19 (0.5%) |
| CKD-EPI_2021_ | 69 | 61.2 ± 15.2 | 27.2 (24.5–31.3) | 46 (0.5%) | 10 (1.2%) | 1 (0.1%) | 1 (1.5%) | 11 (0.3%) |
| EKFC | 94 | 65.9 ± 14.0 | 26.9 (24.6–30.9) | 62 (0.7%) | 12 (1.5%) | 1 (0.1%) | 1 (1.5%) | 18 (0.4%) |
| LMR | 173 | 64.5 ± 13.8 | 27.3 (24.6–32.2) | 114 (1.2%) | 21 (2.6%) | 4 (0.5%) | 1 (1.5%) | 33 (0.8%) |

# Equation abbreviations: MDRD = Modified Diet in Renal Disease; CKD-EPI = Chronic Kidney Disease Epidemiology Collaboration; EKFC = European Kidney Function Consortium; LM = Lund-Malmo.

Reference:

28	Marrington R, MacKenzie F. Variation of eGFR reporting and CKD equations used in the United Kingdom. *Annals of Clinical Biochemistry: International Journal of Laboratory Medicine* 2023; **60**: 328–38.

29	Flamant M, Vidal-Petiot E, Metzger M, *et al.* Performance of GFR estimating equations in African Europeans: Basis for a lower race-ethnicity factor than in African Americans. American Journal of Kidney Diseases. 2013; **62 (1)**: 182–4.

30	Delanaye P, Vidal-Petiot E, Björk J, *et al.* Performance of creatinine-based equations to estimate glomerular filtration rate in White and Black populations in Europe, Brazil and Africa. *Nephrology Dialysis Transplantation* 2023; **38**: 106–18.
